# Dynamical Models Explaining Social Balance and Evolution of Cooperation

**DOI:** 10.1371/journal.pone.0060063

**Published:** 2013-04-25

**Authors:** Vincent Antonio Traag, Paul Van Dooren, Patrick De Leenheer

**Affiliations:** 1 ICTEAM, Université catholiquede Louvain, Louvain-la-Neuve, Belgium; 2 Department of Mathematics, University of Florida, Gainesville, Florida, United States of America; University of Namur, Belgium

## Abstract

Social networks with positive and negative links often split into two antagonistic factions. Examples of such a split abound: revolutionaries versus an old regime, Republicans versus Democrats, Axis versus Allies during the second world war, or the Western versus the Eastern bloc during the Cold War. Although this structure, known as social balance, is well understood, it is not clear how such factions emerge. An earlier model could explain the formation of such factions if reputations were assumed to be symmetric. We show this is not the case for non-symmetric reputations, and propose an alternative model which (almost) always leads to social balance, thereby explaining the tendency of social networks to split into two factions. In addition, the alternative model may lead to cooperation when faced with defectors, contrary to the earlier model. The difference between the two models may be understood in terms of the underlying gossiping mechanism: whereas the earlier model assumed that an individual adjusts his opinion about somebody by gossiping about that person with everybody in the network, we assume instead that the individual gossips with that person about everybody. It turns out that the alternative model is able to lead to cooperative behaviour, unlike the previous model.

## Introduction

**Figure 1:**
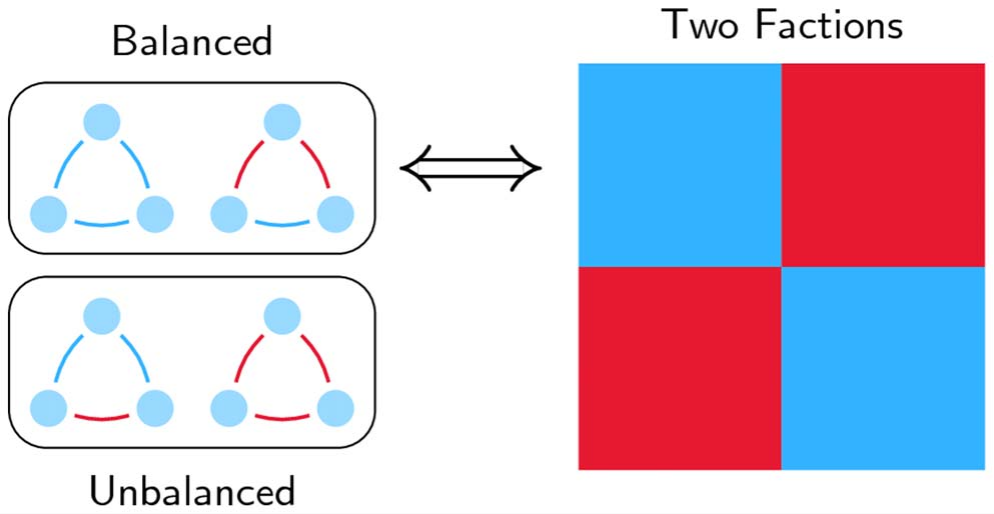
Social Balance. The two upper triads are balanced, while the two lower triads are unbalanced. According to the structure theorem [3], a complete graph can be split into (at most) two opposing factions, if and only if all triads are balanced. This is represented by the coloured matrix on the right, where blue indicates positive entries, and red negative entries.

Why do two antagonistic factions emerge so frequently in social networks? This question was already looming in the 1940s, when Heider [1] examined triads of individuals in networks, and postulated that only balanced triads are stable. A triad is balanced when friends agree in their opinion of a third party, while foes disagree, see Fig. 1. The individuals in an unbalanced triad have an incentive to adjust their opinions so as to reduce the stress experienced in such a situation [2]. Once an adjustment is made, the triad becomes balanced, and the stress disappears.

A decade later, Harary [3] showed that a complete social network splits in at most two factions if and only if all its triads are balanced, see also [4]. Such networks are called (socially) balanced as well. Since then, the focus of much of the research has been on detecting such factions in signed networks [5], [6]. Many signed networks show evidence of social balance, although the split into factions might not be exact, that is, they are only nearly socially balanced [7]–[10].

What has been lacking until fairly recently, are dynamical models that explain *how* social balance emerges. The purpose of this paper is to analyse two such models. One of these models, proposed first in [11], was proved to exhibit social balance in [12]. However, this was done under a restrictive symmetry assumption for the reputation matrix. Here, we continue the analysis of this model and show that it generically does not lead to social balance when the symmetry assumption is dropped. In contrast, we propose a second model that is based on a different underlying gossiping mechanism, and show that it generically does lead to social balance, even when reputations are not symmetric.

Moreover, there is a natural connection between negative links and the evolution of cooperation: we consider positive links as indicating cooperation and negative links as defection. We will show that our alternative model is able to lead to cooperation, whereas the earlier model cannot.

## Earlier Model

Certain discrete-time, stochastic dynamics have been investigated [13], [14], but they exhibit so-called jammed states [15]: no change in the sign of a reputation improves the degree of social balance, as measured by the total number of balanced triads in the network. A surprisingly simple continuous-time model [11] was proved to converge to social balance for certain symmetric initial conditions [12]. The authors assume that the social network is described by a complete graph (everybody is connected to everybody), with weighted links representing reputations that change continuously in time. Let *X* denote the real-valued matrix of the reputations, so that *X_ij_* represents the opinion *i* has about *j*. It is positive whenever *i* considers *j* a friend, and negative if *i* thinks of *j* as an enemy. The network is balanced, if, up to a possible relabelling of the individuals, the sign structure of *X* takes one of two possible block forms:
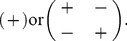
(1)


Changes in the reputations are modelled as follows:
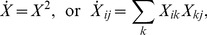
(2)where 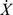 denotes the derivative with respect to time of the matrix *X*. The idea behind this model is that reputations are adjusted based on the outcome of a particular gossiping process. More specifically, suppose that Bob (individual *i*) wants to revise his opinion about John (individual *j*). Bob then asks everybody else in the network what they think of John. If one such opinion *X_kj_* has the same sign as the opinion Bob has about his gossiping partner, i.e. as *X_ik_*, then Bob will increase his opinion about John. But if these opinions differ in sign, then Bob will decrease his opinion about John.

The analysis for symmetric initial conditions *X*(0) = *X^T^* (0) was carried out in [12]: First, *X*(0) is diagonalized by an orthogonal transformation *X*(0) = *U*Λ(0)*U^T^*, where the columns of *U* are orthonormal eigenvectors *u*_1_,…,*u_n_* of *X*(0) so that *UU^T^* = *I_n_*, and Λ(0) is a diagonal matrix whose diagonal entries are the corresponding real eigenvalues λ_1_(0) ≥ λ_2_(0)≥ · ≥ λ*_n_*(0) of *X*(0). Direct substitution of the matrix function *U*Λ(*t*)*U^T^* shows that it is the solution of Eq. 2 with initial condition *X*(0). Here, Λ(*t*) is a diagonal matrix, solving the uncoupled matrix equation 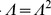 with initial condition Λ(0). The diagonal entries of Λ(*t*) are obtained by integrating the scalar first order equations 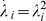:
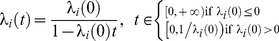
(3)


Hence, the solution *X*(*t*) blows up in finite time if and only if λ_1_(0) >0. Moreover, if λ_1_(0) >0 is a simple eigenvalue, then the solution *X*(*t*), normalized by its Frobenius norm, satisfies:
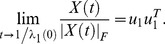
(4)


Assuming that *u*_1_ has no zero entries, and up to a suitable permutation of its components, the latter limit takes one of the forms in Eq. 1. In other words, if the initial reputation matrix is symmetric and has a simple, positive eigenvalue, then the normalized reputation matrix becomes balanced in finite time.

**Figure 2:**
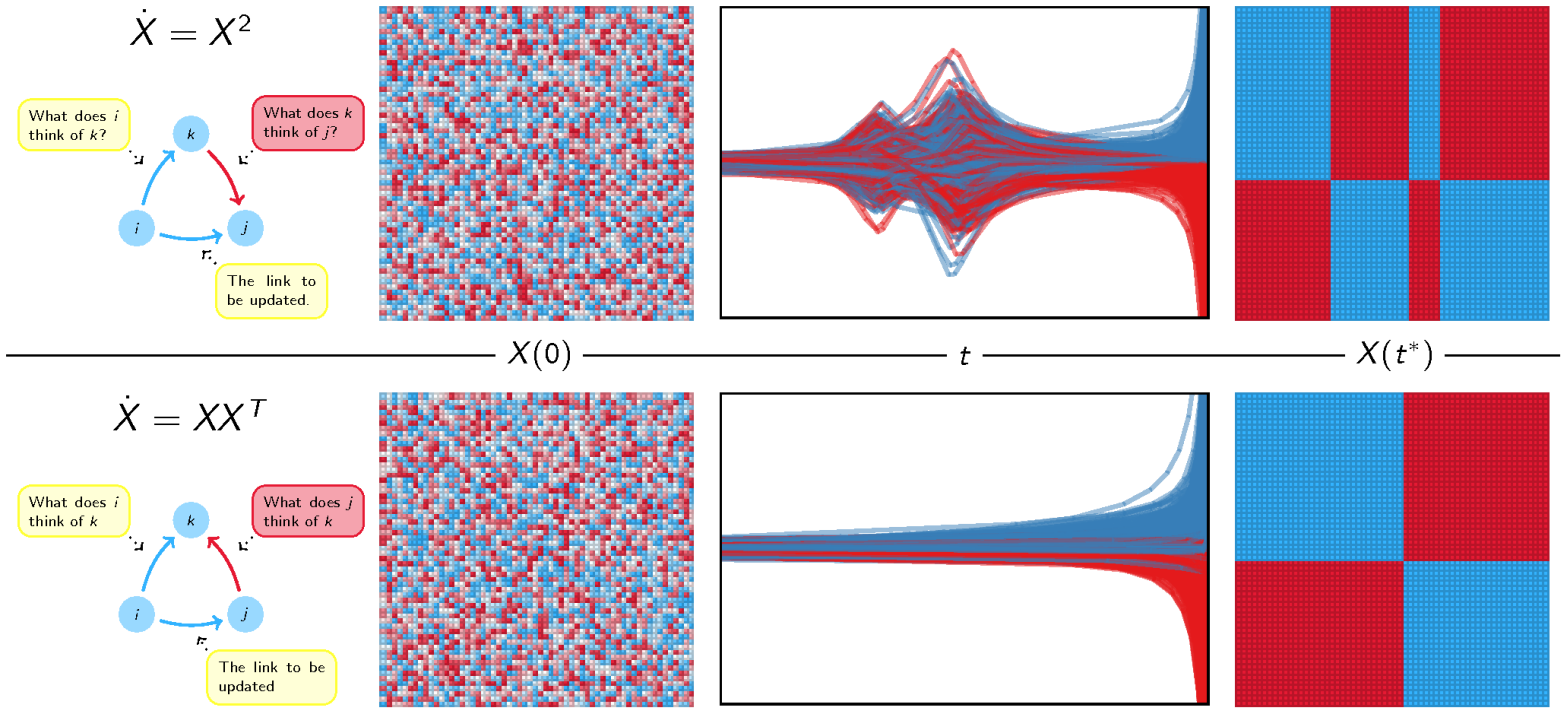
The two models compared. The first row illustrates what happens generically for the model 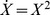, while the second row displays the results for 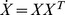. Each row contains from left to right: (1) an illustration of the model; (2) the random initial state; (3) the dynamics of the model; and (4) the final state to which the dynamics converge. Blue indicates positive entries, and red negative entries. Although the first model converges to a rank one matrix, it is not socially balanced. The second model does converge generically to social balance. The small bumps in the dynamics for 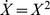 are due to complex eigenvalues that show circular behaviour (see Fig. S1).

Our first main result is that this conclusion remains valid for normal initial conditions, i.e. for initial conditions that satisfy the equality *X*(0)*X^T^* (0) = *X^T^* (0)*X*(0), see SI Text S1, Theorem 2. Whereas the real eigenvalues behave similar to the symmetric case, the complex eigenvalues show circular behaviour, which results in small “bumps” in the dynamics as shown in Fig. 2 (see SI Fig. S1 for more detail). More precisely, if *X*(0) is normal and if λ_1_(0) is a real, positive and simple eigenvalue which is larger than every other real eigenvalue (if any), then the solution *X*(*t*) of Eq. 2 satisfies Eq. 4. Hence, once again, the normalized reputation matrix converges to a balanced state.

Our second main result is that this conclusion does not carry over to the case where *X*(0) is not normal, see SI Text S1, Theorem 3. This general case is analysed by first transforming *X*(0) into its real Jordan-canonical form *J*(0): *X*(0) = *TJ*(0)*T* ^–1^, where *T* consists of a basis of (the real and imaginary parts of) generalized eigenvectors of *X*(0). It can then be shown that the solution *X*(*t*) of Eq. 2 is given by *TJ*(*t*)*T* ^–1^, where *J*(*t*) solves the matrix equation 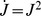, an equation which can still be solved explicitly. Hence, *X*(*t*) can still be determined. It turns out that if *X*(0) has a real, positive and simple eigenvalue λ_1_(0) which is larger than every other real eigenvalue (if any), then the normalized reputation matrix satisfies:
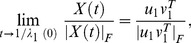
(5)where *u*_1_ and 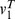 are left and right eigenvectors of *X*(0) respectively, that correspond to the eigenvalue λ_1_(0). If we assume that none of the entries of *u*_1_ and *v*_1_ are zero, then we can always find a suitable permutation of the components of *u*_1_ and *v*_1_ such that they have the following sign structure:



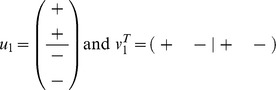



Consequently, in general, the matrix limit in Eq. 5 has the sign structure:
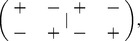
as illustrated in Fig. 2. Clearly, this configuration doesn't correspond to social balance any longer.

## Alternative Model

Let us briefly reconsider the gossiping process underlying model 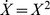. In our example of Bob and John, the following happens. Bob asks others what they think of John. Bob takes into account what he thinks of the people he talks to, and adjusts his opinion of John accordingly. An alternative approach is to consider a type of homophily process [16]–[18]: people tend to befriend people who think alike. When Bob seeks to revise his opinion of John, he talks to John about everybody else (instead of talking to everybody else about John). For example, suppose that Bob likes Alice, but that John dislikes her. When Bob and John talk about Alice, they notice they have opposing views about her, and as a result the relationship between Bob and John deteriorates. On the other hand, should they share similar opinions about Alice, their relationship will improve. Thus, our alternative model for the update law of the reputations is:
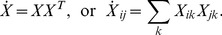
(6)


Although there apparently is only a subtle difference in the gossiping processes underlying the models in Eq. 2 and 6, these models turn out to behave quite differently, as we discuss next.

Our third main result is that for generic initial conditions, the normalized solution of system Eq. 6 converges to a socially balanced state in finite time. To show this, we decompose the solution *X*(*t*) into its symmetric and skew-symmetric parts: *X*(*t*) = *S*(*t*)+ *A*(*t*), where *S*(*t*) = *S^T^*(*t*) and *A*(*t*) = –*A^T^* (*t*). Since 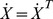, the skew-symmetric part remains constant, and therefore *A*(*t*) = *A*(0) ≡ *A*_0_. The symmetric part then obeys the matrix Riccati differential equation 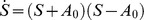. We introduce 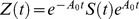 to eliminate the linear terms in this equation, and obtain
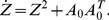
(7)


The latter matrix Riccati differential equation can be integrated, yielding the solution *Z*(*t*) explicitly, and hence *S*(*t*), as well as *X*(*t*), can be calculated.

It turns out that if *A*_0_ ≠ 0, then *X*(*t*) always blows up in finite time. Moreover, using a perturbation argument, it can be shown there is a dense set of initial conditions *X*(0) such that the normalized solution of Eq. 6 converges to
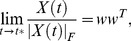
(8)for some vector *w*, as *t* approaches the blow-up time t*, see SI Text S1, Theorem 5. If *w* has no zero entries, this implies that the normalized solution becomes balanced in finite time. Hence, the alternative model in Eq. 6 generically evolves to social balance, see Fig. 2.

## Evolution of Cooperation

Positive and negative links have a natural interpretation in the light of cooperation: positive links indicate cooperation and negative links indicate defection. There is then also a natural motivation for the alternative model in terms of cooperation. Again, suppose Bob wants to revise his opinion of John. For Bob it is important to know whether John is cooperative in order to determine whether he should cooperate with John or not. So, instead of asking Alice whether she has cooperated with John, Bob would like to know whether John has cooperated with her. In other words, Bob is not interested in *X_kj_* but in *X_jk_*, consistent with Eq. 6, illustrated in Fig. 2. This is also what is observed in studies on gossip: it often concerns what others did, not what one thinks of others [19], [20].

**Figure 3:**
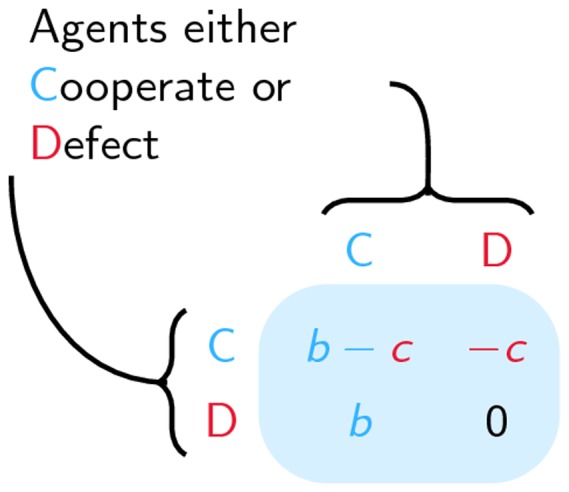
Prisoner's Dilemma. Both players have the option to either Cooperate or Defect. Whenever an agent cooperates, it costs him *c* while his partners receives a benefit *b*>*c*, leading to the indicated payoffs.

Indeed gossiping seems crucial in explaining the evolution of human cooperation through indirect reciprocity [21]. It has even been suggested that humans developed larger brains in order to gossip, so as to control the problem of cooperation through social interaction [22]. In general, the problem is that if defection allows individuals to gain more, why then do individuals cooperate? This is usually modelled in the form of a prisoner's dilemma, in which each agent has the possibility to give his partner some benefit *b* at some cost *c*<*b*. So, if an agent's partner cooperates (he gives the agent *b*) but the agent doesn't cooperate (he doesn't pay the cost *c*) his total payoff will be *b*. Considering the other possibilities results in the payoff matrix detailed in Fig. 3.

Irrespective of the choice of the other player, it is better to defect in a single game. Suppose that the second player cooperates. Then if the first player cooperates he gains *b* – *c*, while if he defects he gains *b*, so defecting is preferable. Now suppose that the second player defects. The first player then has to pay *c*, but doesn't have to pay anything when defecting. So indeed, in a single game, it is always better to defect, yet the payoff is higher if both cooperate, whence the dilemma.

In reality, we do observe cooperation, and various mechanisms for explaining the evolution of cooperation have been suggested [23], such as kin selection [24], [25], reciprocity [26] or group selection [27]. Humans have a tendency however to also cooperate in contexts beyond kin, group or repeated interactions. It is believed that some form of indirect reciprocity can explain the breadth of human cooperation [21]. Whereas in direct reciprocity the favour is returned by the interaction partner, in indirect reciprocity the favour is returned by somebody else, which usually involves some reputation. It has been theorized that such a mechanism could even form the basis of morality [28]. Additionally, reputation (and the fear of losing reputation) seems to play an important role in maintaining social norms [29]–[31].

In general, the idea is the following: agents obtain some good reputation by helping others, and others help those with a good reputation. Initially a strategy known as image scoring was introduced [32]. Shortly after, it was argued that a different strategy, known as the standing strategy, should actually perform better [33], although experiments showed people tend to prefer the simpler image scoring strategy [34]. This led to more systematic studies of how different reputation schemes would perform [35]–[37]. Although much research has been done on indirect reciprocity, only few theoretical works actually study how gossiping shapes reputations [38], [39]. Nonetheless, most studies (tacitly) assume that reputations are shaped through gossip. Additionally, it was observed experimentally that gossiping is an effective mechanism for promoting cooperation [40]–[42].

Moreover, these reputations are usually considered as objective. That is, all agents know the reputation *X_j_* of some agent *j*, and all agents have the same view of agent *j*. Private reputations–so that we have *X_ij_*, the reputation of *j* in the eyes of *i*–have usually been considered by allowing a part of the population to “observe” an interaction, and update the reputation accordingly. If too few agents are allowed to “observe” an interaction, the reputations *X_ij_* tend to become uncorrelated and incoherent. This makes reputation unreliable for deciding whether to cooperate or defect. The central question thus becomes how to model private reputations such that they remain coherent and reliable for deciding whether to cooperate or not.

Dynamical models of social balance might provide an answer to this question. Although it allows to have private reputations–that is *X_ij_*–the dynamics could also lead to some coherence in the form of social balance. In addition, it models more explicitly the gossiping process, commonly suggested to be the foundation upon which reputations are forged.

## Simulation Results

The reputations of the agents are determined by the dynamics of the two models. We call agents using 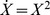 dynamics type A, and those using 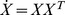 dynamics type B. We assume that agent *i* cooperates with *j* whenever *X_ij_* >0 and defects otherwise. Agents reproduce proportional to their fitness, determined by their payoff. Agents that do well (have a high payoff) have a higher chance of reproduction, and we are interested in knowing the probability that a certain type becomes fixated in the population (i.e. takes over the whole population), known as the fixation probability *ρ*. All simulations start off with an equal amount of agents, so if some type wins more often than his initial relative frequency, it indicates it has an evolutionary advantage. For the results presented here this comes down to *ρ* >1/2. More details on the simulations are provided in the Materials and Methods section at the end of the paper.

**Figure 4:**
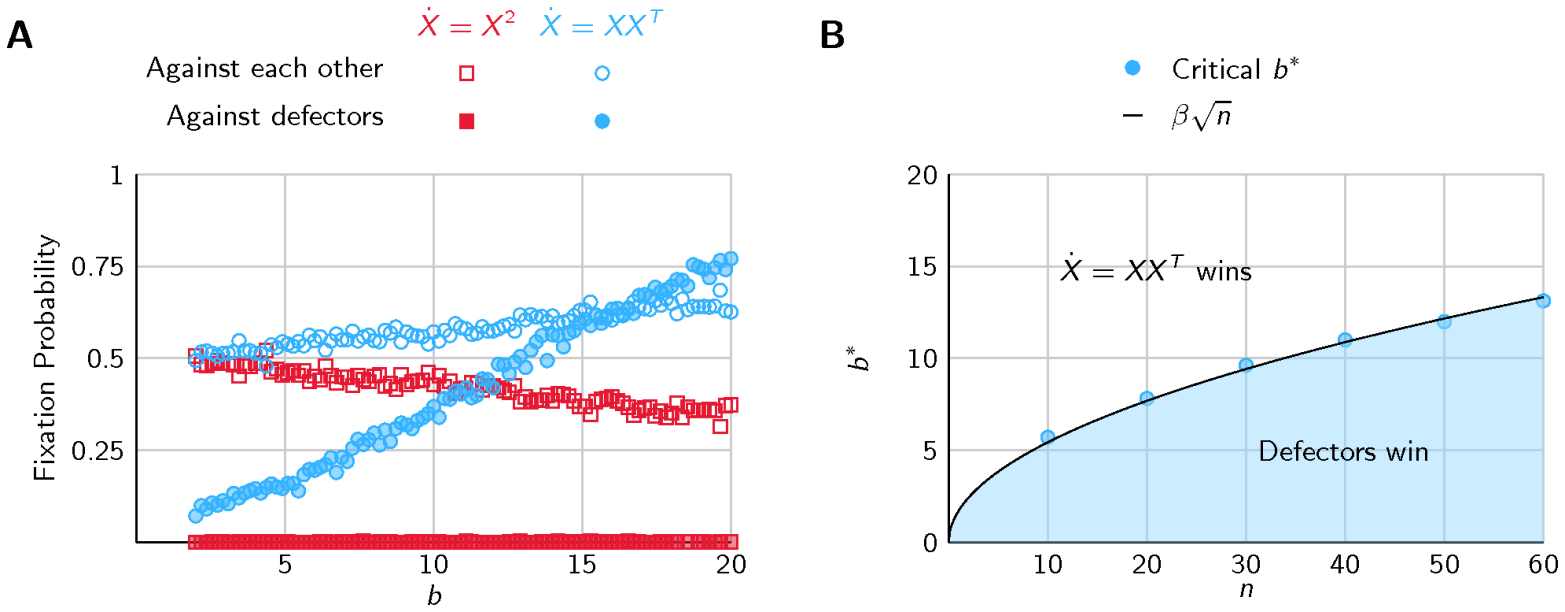
Evolution of Cooperation. (A) The fixation probability (probability to be the sole surviving species) is higher for model 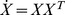 than 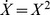. This implies that the model 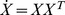 is more viable against defectors, and has an evolutionary advantage compared to 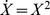. (B) The point b* at which the model 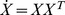 has an evolutionary advantage against defectors (i.e. the fixation probability *ρ* >1/2) depends on the number of agents *n*. The condition for the model 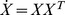 to defeat defectors can be approximated by 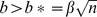, with *β* ≈ 1.72.

The results are displayed in Fig. 4 using a normalized cost of *c*  = 1 (the ratio *b*/*c* drives the evolutionary dynamics, see Materials and Methods and [23]). When directly competing against each other, type B has an evolutionary advantage (its fixation probability *ρ_B_* >1/2) compared to type A, already for relatively small benefits. When each type is playing against defectors (agents that always defect), type A seems unable to defeat defectors (*ρ_A_* <1/2) for any *b* <20, while type B performs quite well against them. When all three types are playing against each other results are similar (see SI Fig. S2). When varying the number of agents, the critical benefit b* at which type B starts to have an evolutionary advantage changes (i.e. where the fixation probability *ρ_B_*  = 1/2). For *b*>b* agents using the model 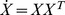 have a higher chance to become fixated, while for *b*<b* defectors tend to win. The inequality for type B to have an evolutionary advantage can be relatively accurately approximated by 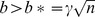 where *γ* is estimated to be around *γ* ≈ 1.72±0.037 (95% confidence interval). Varying the intensity of selection does not alter the results qualitatively (see SI Fig. S3). Summarizing, type B is able to lead to cooperation and defeats type A. Based on these results, if a gossiping process evolved during the course of human history in order to maintain cooperation, the model 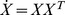 seems more likely to have evolved than 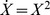. For smaller groups a smaller benefit is needed for the model 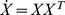 to become fixated. This dependence seems to scale only as 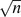, so that larger groups only need a marginally larger benefit in order to develop cooperation.

### Conclusion

To conclude, we have shown that the alternative model 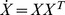 generically converges to social balance, whereas the model 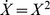 did not. The current models exhibit several unrealistic features, we would like to address: (1) an all-to-all topology; (2) dynamics that blow-up in finite time; and (3) homogeneity of all agents. Although most of these issues can be addressed by specifying different dynamics, the resulting models are much more difficult to analyse, thereby limiting our understanding. Although the two models are somewhat simple, they are also tractable, and what we lose in truthfulness, we gain in deeper insights: in simplicity lies progress. Our current analysis offers a quite complete understanding, and we hope it provides a stepping stone to more realistic models, which we would like to analyse in the future.

The difference between the two models can be understood in terms of gossiping: we assume that people who wish to revise their opinion about someone talk to that person about everybody else, while the earlier model assumed that people talk about that person to everybody else. Both gossiping and social balance are at the centre of many social phenomena [22], [29], [43], [44], such as norm maintenance [30], stereotype formation [45] and social conflict [46]. For example, a classic work [29] on the established and outsiders found that gossiping was the fundamental driving force for the maintenance of the cohesive network of the established at the exclusion of the outsiders. Understanding how social balance may emerge might help to understand the intricacies of these social phenomena.

Moreover, in light of the evolution of cooperation it appears that agents using 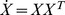 dynamics perform well against defectors, and have an evolutionary advantage compared to agents using 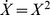 dynamics. Contrary to other models of indirect reciprocity, not everybody might end up cooperating with everybody, and the population may split into two groups. This provides an interesting connection between social balance theory, gossiping and the evolution of cooperation. Our results improve our understanding of gossiping as a mechanism for group formation and cooperation, and as such contributes to the study of indirect reciprocity.

## Materials and Methods

In the simulations of the evolution of cooperation, the dynamics consist of two parts: (1) the interaction dynamics within each generation; and (2) the dynamics prescribing how the population evolves from generation to generation.

### Interaction Dynamics

We include three possible types of agents in our simulations:

**Type A** uses 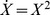 dynamics,

**Type B** uses 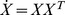 dynamics, and

**Defectors** have trivial reputation dynamics 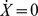, with negative constant reputations.

We can decompose the reputation matrix *X*(*t*) accordingly into three parts:
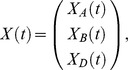
where *X_A_*(*t*) are the reputations of all agents in the eyes of agents of type A, *X_B_*(*t*) for type B and *X_D_*(*t*) for defectors. The reputations *X_A_*(0) and *X_B_*(0) are initialized from a standard Gaussian distribution. The initial reputation for *X_D_*(0) will be set to a fixed negative value. To be clear, *X_D_*(0) is the reputation of all other agents in the eyes of defectors, which is negative initially. The initial reputation of the defectors themselves is of course not necessarily negative initially. For the results displayed here we have used *X_D_*(0) = −10, but results remain by and large the same when varying this parameter, as long as it remains sufficiently negative.

Since we are dealing with continuous dynamics in this paper, we assume all agents are involved in infinitesimally short games at each time instance *t*. Each agent *i* may choose to either cooperate or defect with another agent *j*, and this decision may vary from one agent to the next. For agents of type A and type B the decision to cooperate is based on the reputation: they defect whenever *X_ij_*(*t*) ≤ 0 and cooperate whenever *X_ij_*(*t*) >0. We define the cooperation matrix *C*(*t*) accordingly.
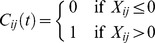



Defectors will simply always defect. Whenever an agent *i* cooperates with *j* the latter receives a payoff of *b* at a cost of *c* to agent *i*. We integrate this payoff over all infinitesimally short games from time 0 to time t*, which can be represented as
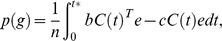
where *e* = (1,…,1) the vector of all ones for a certain generation *g*.

### Evolutionary Dynamics

We have simulated the evolution of cooperation for *n*  = 10,20,…,60 agents, which stays constant throughout evolution. We consider four different schemes for initializing the first generation:

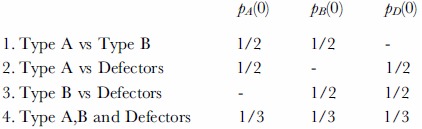



Here *p_A_*(0),*p_B_*(0) and *p_D_*(0) are respectively the proportion of agents of type A, type B and defectors in the first generation. We use the vector *T_i_*(*g*) ∈ {*A*, *B*, *D*} to denote the type of agent *i* in generation *g*, so that *T_i_*(*g*) = *A* if agent *i* is a type A player, *T_i_*(*g*) = *B* for a type B player, and *T_i_*(*g*) = *D* for a defector. We are interested in estimating the probability that a single type takes over the whole population, known as the fixation probability *ρ_A_*, *ρ_B_* and *ρ_D_* for the three different types. If a type has no evolutionary advantage, it is said to be evolutionary neutral, and in that case its fixation probability is equal to its initial frequency, e.g. for type A *ρ_A_* = *p_A_*(0).

We will keep the population constant at the initial *n*, and simply choose *n* new agents according to their payoff for the next generation. This can be thought of as choosing *n* times one of the *n* agents in the old generation for reproduction. Let *φ_i_* denote the probability that an agent is selected for reproduction, which we define as
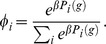



Since we are only interested in the number of agents of a certain type, we can also gather all payoffs for the same type of agents, and write
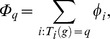
where *q* ∈ {*A*, *B*, *D*} represents the type of agent. The probability to select a type A agent, a type B agent or a defector is then respectively Φ*_A_*, Φ*_B_* and Φ*_D_*. In the next generation, the probability that agent *i* is of a specific type *q* can then be written as



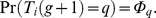



This evolutionary mechanism can be seen as a Wright-Fisher process [47] with fitnesses 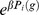. It is well known that this process converges faster than a Moran birth-death process, since it essentially takes *n* time steps in a Moran process to reproduce the effect of one time step in a Wright-Fisher process [47]. Because of the high computational costs (solving repeatedly a non-linear system of differential equations of size *n* ^2^), this process is preferable.

Higher *β* signifies higher selective pressure, and leads to a higher reproduction of those with a high payoff, and in the case that *β* → ∞ only those with the maximum payoff reproduce. On the other hand, for *β* → 0 this tends to the uniform distribution *φ_i_*  = 1/*n*, where payoffs no longer play any role. We have used *β*  = 0.5 for the low selective pressure, *β*  = 5 for the high selective pressure, reported in SI Fig. S3. For the results in the main text we have used *β*  = 1.

For an evolutionary neutral selection in where all *P_i_*(*g*) = *P* are effectively the same, *β* has no effect, and *φ_i_*  = 1/*n*. Notice that if we rescale *P_i_*(*g*) by 1/*c* so that the payoff effectively becomes
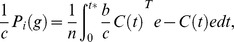
and we rescale *β* by *c*, then the reproduction probabilities remain unchanged. Hence, only the ratio *b*/*c* effectively plays a role up to a rescaling of the intensity of selection. Since the point at which the evolution is neutral (i.e. *ρ* equals the initial proportional frequency), is independent of *β*, this point will only depend on the ratio *b*/*c*. So, we normalized the cost *c*  = 1. To verify this, we also ran additional simulations with different costs, which indeed gave the same results.

We stop the simulation whenever one of the types becomes fixated in the population. With *fixation* we mean that all other types have gone extinct, and only a single type remains. If no type has become fixated after 1,000 generations, we terminate the simulation and count as winner the most frequent type. This almost never happens, and the simulation usually stops after a relatively small number of generations.

In total, we repeat this process 1,000 times for the results in the main text, and for the low (*β*  = 0.5) and high (*β*  = 5) selective pressure 100 times. This means that we run the evolutionary dynamics until one of the types has become fixated, and we record which type has “won”. After that, we again start from the first generation, and run until fixation, and repeat this. Finally, we calculate how many rounds a type has “won” compared to the total number of rounds, which yields the fixation probability *ρ*.

## Supporting Information

Figure S1 **Phase portrait of system S12-S13.** Circular orbits in the upper half plane (*a* >0) are traversed counter clockwise, whereas circular orbits in the lower half plane (*a* <0) are traversed clockwise.
(TIFF)

Figure S2 **Results including type A, B and defectors.**
(TIFF)

Figure S3 **Results different intensities of selection.**
(TIFF)

Text S1 **Proofs and details of statements in the main paper.**
(PDF)
