# Supplementary figures and images for: Dynamical Models Explaining Social Balance and Evolution of Cooperation
Source: PLoS One. 2013 Apr 25;8(4):e60063. doi: 10.1371/journal.pone.0060063 (PMC3636264; doi:10.1371/journal.pone.0060063)

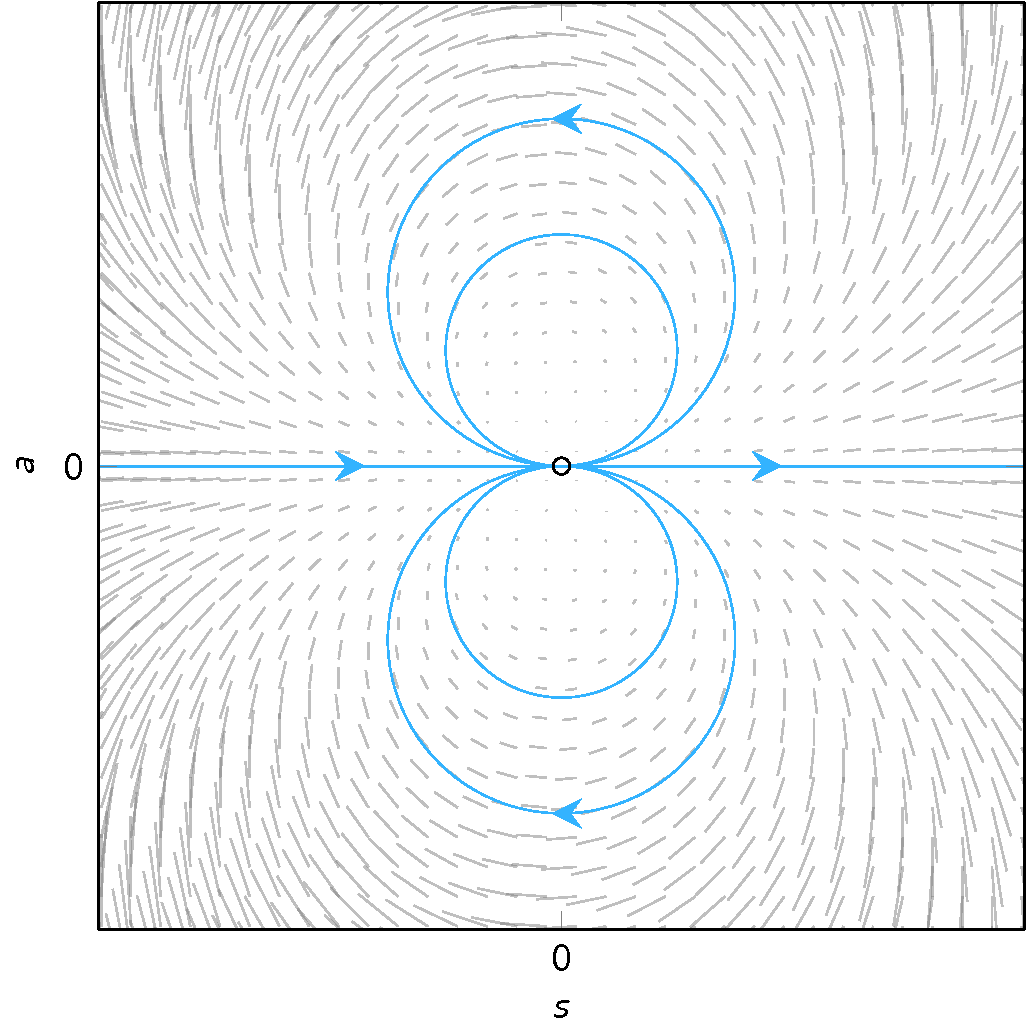

Supplement: Figure S1 — Phase portrait of system S12-S13. Circular orbits in the upper half plane (a >0) are traversed counter clockwise, whereas circular orbits in the lower half plane (a <0) are traversed clockwise. (TIFF) [file pone.0060063.s001.tif]

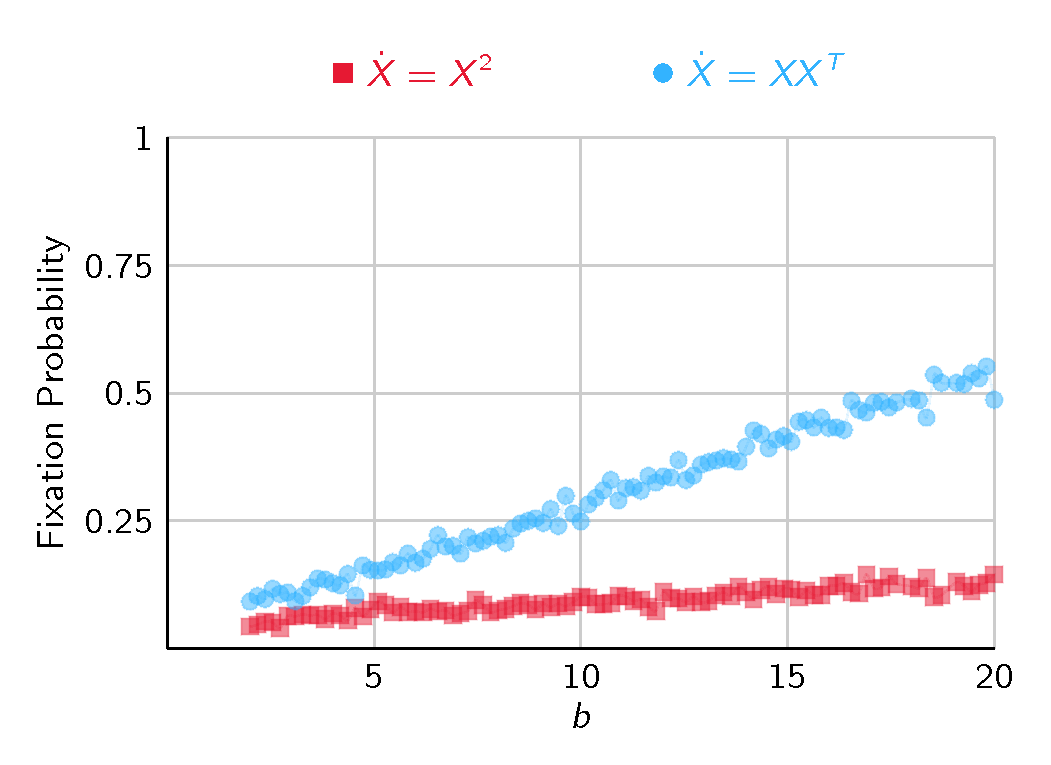

Supplement: Figure S2 — Results including type A, B and defectors. (TIFF) [file pone.0060063.s002.tif]

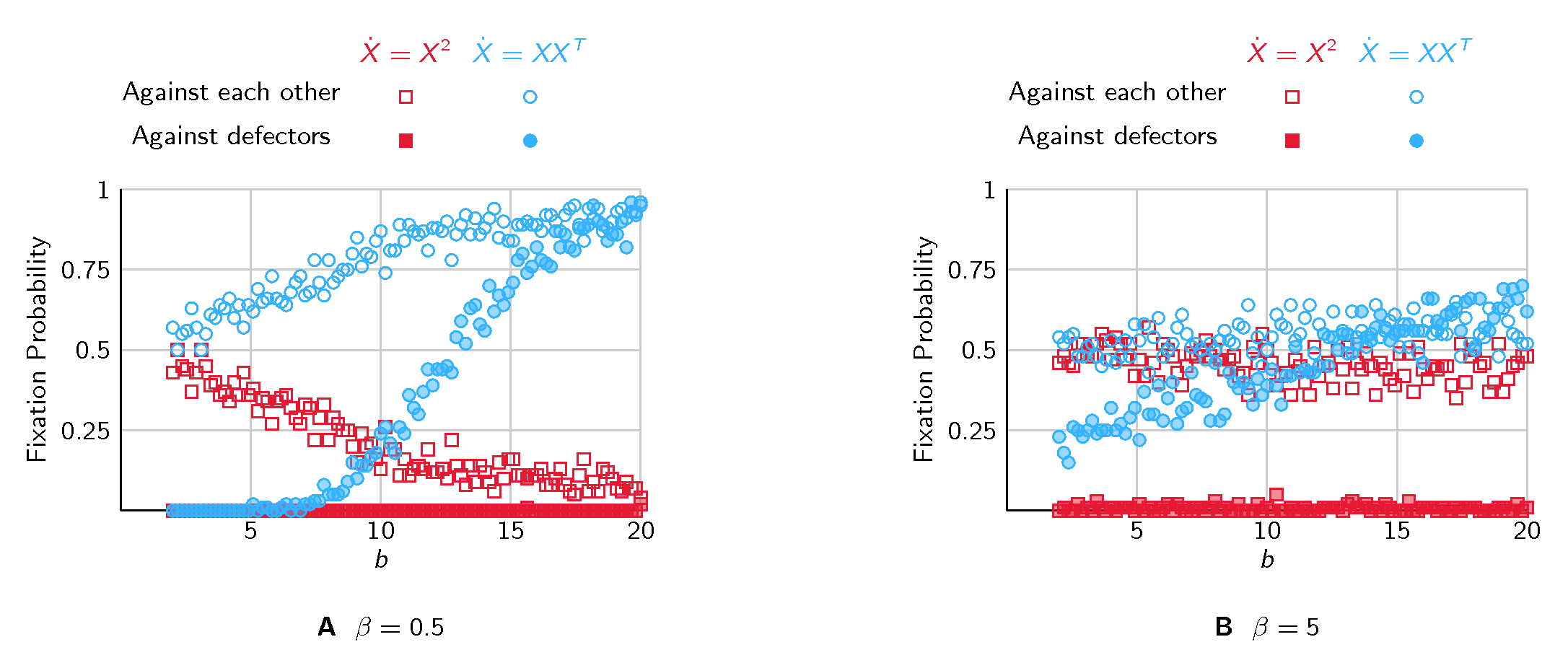

Supplement: Figure S3 — Results different intensities of selection. (TIFF) [file pone.0060063.s003.tif]
