# Supplementary material for: Dynamical Models Explaining Social Balance and Evolution of Cooperation
Source: PLoS One. 2013 Apr 25;8(4):e60063. doi: 10.1371/journal.pone.0060063 (PMC3636264; doi:10.1371/journal.pone.0060063)
Supplement: Text S1 — Proofs and details of statements in the main paper. (PDF) [file pone.0060063.s004.pdf]

Supporting Information
Dynamical Models Explaining Social Balance and Evolution of Cooperation

Vincent Antonio Traag[1,*], Paul Van Dooren[1], Patrick De Leenheer[2]
1 ICTEAM, Université catholique de Louvain, 1348, Louvain-la-Neuve, Belgium
2 Department of Mathematics, University of Florida, FL 32601, Gainesville, United States
∗ E-mail: Vincent.Traag@uclouvain.be

Preliminaries

We investigate matrix differential equations of the form $\dot{X} = F(X, X^T)$, where X is a real $n \times n$ matrix, and F is a one of two specific, smooth functions. These functions are such that it turns out to be advantageous to consider the dynamics of the symmetric and skew-symmetric parts of X. Recall that $\mathbb{R}^{n\times n} = \mathcal{S} \oplus \mathcal{A}$, where $\mathcal{S}$ is the linear subspace of real symmetric matrices, and $\mathcal{A}$ the linear subspace of skew-symmetric matrices. Thus, given any $X \in \mathbb{R}^{n\times n}$, we can find unique symmetric $S \in \mathcal{S}$ and skew-symmetric $A \in \mathcal{A}$ such that $X = S + A$. More explicitly, $S = (X + X^T)/2$ and $A = (X - X^T)/2$. Moreover, using the inner product $\langle X, Y\rangle = \mathrm{tr}(XY^T)$, there holds that

$$\mathcal{A}^\perp = \mathcal{S}. \tag{S1}$$

The norm induced by this inner product is the Frobenius norm $|X|_F = (\mathrm{tr}(XX^T))^{\frac{1}{2}}$. Recall that the Frobenius norm is unitarily invariant, i.e. if U is orthogonal (i.e. $UU^T = I_n$), then

$$|UXU^T|_F = |X|_F. \tag{S2}$$

We denote by I_n the $n \times n$ identity matrix, and by J_n a specific skew symmetric matrix:

$$J_n = \begin{pmatrix} 0 & I_{n/2} \\ -I_{n/2} & 0 \end{pmatrix}, n \text{ even}. \tag{S3}$$

For all other linear algebra related terminology and properties we refer to [1].

We briefly review two key ingredients of Heider's (static) theory on social balance, namely those of a *balanced triangle* and a *balanced network*:

Definition 1. *A triangle of (not necessarily distinct) agents i, j and k is called balanced if*

$$X_{ij}X_{ik}X_{kj} > 0. \tag{S4}$$

A network is said to be balanced if all triangles of agents in the network are balanced.

It turns out that a balanced network takes on a specific structure, in that at most 2 factions emerge, where members within each faction have positive opinions about each other, but members in different factions have negative opinions about each other. This result is known as the Structure Theorem [2, 3]:

Theorem 1 (Structure Theorem in [2,3]). *Let X represent a balanced network. Then up to a permutation of agents, the matrix X has the following sign structure:*

$$(+) \quad or \quad \begin{pmatrix} + & - \\ - & + \end{pmatrix}.$$

Conversely, if, up to permutation, X has one of these structures, then it represents a balanced network.

Notice that the same theorem holds irrespective of any permutation of i,j and k in definition 1.

Equation $\dot{X} = X^2$

Consider the model studied numerically in [4] and analysed for symmetric initial conditions in [5]:

$$\dot{X} = X^2, X(0) = X_0, \tag{S5}$$

where each X_{ij} is real-valued and denotes the opinion agent i has about agent j. Positive values mean that agent i thinks favourably about j, whereas negative values mean that i thinks unfavourably about j. More explicitly, model S5 can also be written entrywise:

$$\dot{X}_{ij} = \sum_k X_{ik} X_{kj}. \tag{S6}$$

The basic question in this context is whether or not the solutions of S5 evolve towards a state which corresponds to a balanced network.[1]

Normal initial condition

We start by defining

$$\mathcal{N} = \{X \in \mathbb{R}^{n\times n} | XX^T = X^T X\},$$

the set of real, normal matrices. Notice that if X belongs to $\mathcal{N}$ then so does X^2, hence the set $\mathcal{N}$ is invariant for $\dot{X} = X^2$.

Recall that normal matrices are (block)-diagonalisable with blocks of size at most 2 by an orthogonal transformation: if $X_0 \in \mathcal{N}$, then

$$U^T X_0 U = \Lambda_0, \tag{S7}$$

where Λ_0 consists of real 1×1 scalar blocks A_i and real 2×2 blocks $B_j = \alpha_j I_2 + \beta_j J_2$ with $\beta_j \neq 0$.

Note that if $\Lambda(t)$ is the solution to the initial value problem $\dot{\Lambda} = \Lambda^2$, $\Lambda(0) = \Lambda_0$, then $X(t) := U\Lambda(t)U^T$ is the solution to Eq. S5. This shows it is sufficient to solve system S5 in case of scalar X or in case of a specific, 2×2, normal matrix X. The scalar case is easy to solve: the solution of $\dot{x} = x^2$, $x(0) = x_0$, is

$$x(t) = \frac{x_0}{1 - x_0 t}, \tag{S8}$$

which is easily verified, so we turn to the 2×2 case by considering:

$$\dot{X} = X^2, \;\; X(0) = \alpha I_2 + \beta J_2, \;\text{ where } \beta > 0. \tag{S9}$$

Lemma 1. *The forward solution $X(t)$ of S9 is defined for all $t \in [0, +\infty)$, and*

$$\lim_{t\to+\infty} X(t) = 0 \;\textit{and}\; \lim_{t\to+\infty} \frac{X(t)}{|X(t)|_F} = -\frac{\sqrt{2}}{2} I_2.$$

Proof. Let $X_0 = S_0 + A_0$, $S_0 = \alpha I_2$ and $A_0 = \beta J_2$ where J_2 is as defined in Eq. S3. Then the solution $X(t)$ of S9 can be decomposed as $S(t) + A(t)$, where

$$\dot{S} = S^2 + A^2, \;\; S(0) = S_0, \tag{S10}$$

$$\dot{A} = AS + SA, \;\; A(0) = A_0. \tag{S11}$$

[1] A minor technical issue is that the solution $X(t)$ of S5 often blows up in finite time $\bar{t}$ as we shall see later. To resolve this problem we investigate the sign pattern of the matrix limit $\lim_{t\to\bar{t}} X(t)/|X(t)|_F$ instead, and say that the network evolves to a balanced state, if this matrix limit is balanced.

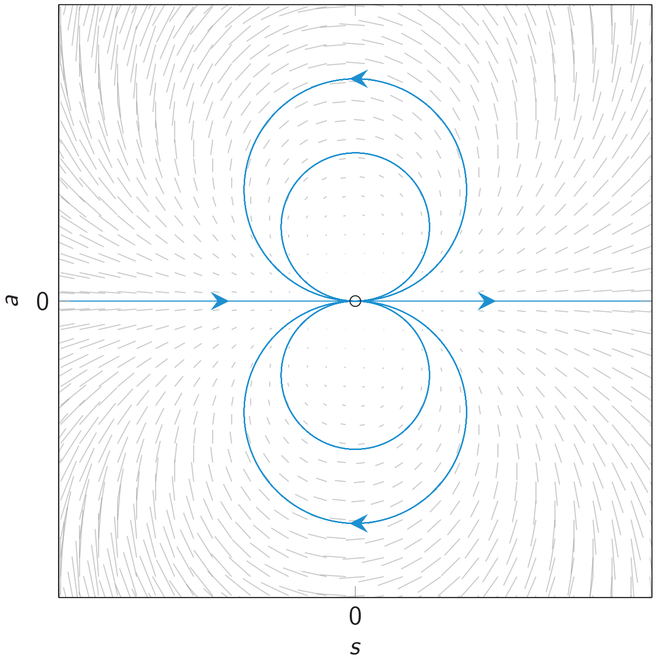

Figure S1. Phase portrait of system S12-S13. Circular orbits in the upper half plane ($a > 0$) are traversed counter clockwise, whereas circular orbits in the lower half plane ($a < 0$) are traversed clockwise.

Note that S10 is a matrix Riccati differential equation with the property that the set $\mathcal{L} := \{sI_2 + aJ_2 | s,\ a \in \mathbb{R}\}$, is an invariant set under the flow. Therefore it suffices to solve the scalar Riccati differential equation corresponding to the dynamics of the scalar coefficients s and a:

$$\dot{s} = s^2 - a^2,\ s(0) = \alpha, \tag{S12}$$

$$\dot{a} = 2as,\ a(0) = \beta, \tag{S13}$$

whose solution is given implicitly by:

$$s^2 + \left(a - \frac{1}{2c}\right)^2 = \left(\frac{1}{2c}\right)^2 \text{ if } c \neq 0,$$

where c is an integration constant. So, the orbits form circles which are centred at $(0, 1/2c)$ and pass through $(0, 0)$, and by $a = 0$ if $c = 0$. The phase portrait of system S12-S13 is illustrated in Fig. S1.

All solutions $(s(t), a(t))$ of system S12-S13, not starting on the s-axis, converge to zero as $t \to +\infty$, and approach the origin in the second quadrant for solutions in the upper-half-plane, and in the third quadrant for solutions in the lower-half-plane. Moreover, since the s-axis is the tangent line to every circular orbit at the origin, the slopes $a(t)/s(t)$ converge to 0 along every solution $\lim_{t\to+\infty} a(t)/s(t) = 0$. Consequently, the forward solution $X(t)$ of S9 satisfies:

$$\lim_{t\to+\infty} X(t) = \lim_{t\to+\infty} s(t)I_2 + a(t)J_2 = 0,$$

and

$$\lim_{t\to+\infty} \frac{X(t)}{|X(t)|_F} = -\frac{\sqrt{2}}{2} I_2.$$

□

Combining the solution for the scalar and 2×2 case yields our main result in the normal case:

Theorem 2. *Let $X_0 \in \mathcal{N}$, and let (U, Λ_0) be as in Eq. S7. Define*

$$\bar{t}_i = \begin{cases} 1/a_i & \text{if } a_i > 0 \\ +\infty & \text{if } a_i \leq 0 \end{cases} \quad \text{for all } i = 1, \ldots, k,$$

and let $\bar{t} = \min_i \bar{t}_i$. Then the forward solution $X(t)$ of S5 is defined for $[0, \bar{t})$.

If there is a unique $i^* \in \{1, \ldots, k\}$ such that $\bar{t} = \bar{t}_{i^*}$ is finite, then

$$\lim_{t \to \bar{t}_{i^*}-} \frac{X(t)}{|X(t)|_F} = U_{i^*} U_{i^*}^T,$$

where U_{i^*} is the i^*th column of U, an eigenvector corresponding to eigenvalue a_{i^*} of X_0.

Proof. Consider the initial value problem:

$$\dot{\Lambda} = \Lambda^2, \ \Lambda(0) = \Lambda_0.$$

Its solution is given by

$$\Lambda(t) = \begin{pmatrix} \frac{a_1}{1-a_1 t} & \ldots & 0 & 0 & \ldots & 0 \\ \vdots & \ddots & \vdots & \vdots & \ddots & \vdots \\ 0 & \ldots & \frac{a_k}{1-a_k t} & 0 & \ldots & 0 \\ 0 & \ldots & 0 & X_1(t) & \ldots & 0 \\ \vdots & \ddots & \vdots & \vdots & \ddots & \vdots \\ 0 & \ldots & 0 & 0 & \ldots & X_l(t) \end{pmatrix},$$

where for all $j = 1, \ldots, l$, $X_j(t)$ is the forward solution of S9, which is defined for all t in $[0, +\infty)$, and converges to 0 as $t \to +\infty$ by Lemma 1.

This clearly shows that $\Lambda(t)$ is defined in forward time for t in $[0, \bar{t})$. Since the solution of S5 is given by $X(t) = U\Lambda(t)U^T$, $X(t)$ is also defined in forward time for t in $[0, \bar{t})$. It follows from S2 that

$$\frac{X(t)}{|X(t)|_F} = U \frac{\Lambda(t)}{|\Lambda(t)|_F} U^T.$$

If $i^* \in \{1, \ldots, k\}$ is the unique value such that $\bar{t} = \bar{t}_{i^*}$, then using S2:

$$\lim_{t \to \bar{t}_i^*} \frac{X(t)}{|X(t)|_F} = U \lim_{t \to \bar{t}_i^*} \frac{\Lambda(t)}{|\Lambda(t)|_F} U^T = U e_{i^*} e_{i^*}^T U^T = U_{i^*} U_{i^*}^T,$$

where e_{i^*} denotes the i^*th standard unit basis vector of $\mathbb{R}^n$. □

Theorem 2 provides a sufficient condition guaranteeing that social balance in the sense of definition 1 is achieved. If X_0 has a simple, positive, real eigenvalue a_{i^*}, and if no entry of the eigenvector U_{i^*} is zero, then the network becomes balanced. Indeed, there holds that, up to a permutation of its entries, the sign pattern of the eigenvector U_{i^*} is either:

$$U_{i^*} = \left(+\right) \text{ or } \left(-\right) \implies U_{i^*} U_{i^*}^T = \left(+\right),$$

or

$$U_{i^*} = \begin{pmatrix} + \\ - \end{pmatrix} \implies U_{i^*} U_{i^*}^T = \begin{pmatrix} + & - \\ - & + \end{pmatrix}.$$

In either case, Theorem 1 implies that the normalized state of the system becomes balanced in finite time.

Generic initial condition

Although Theorem 2 provides a sufficient condition for the emergence of social balance, it requires that the initial condition X_0 is normal. But the set $\mathcal{N}$ of normal matrices has measure zero in the set of all real $n \times n$ matrices, and thus the question arises if social balance will arise for non-normal initial conditions as well. We investigate this issue here, and will see that generically, social balance is not achieved.

If X_0 is a general real $n \times n$ matrix, we can put it in real Jordan canonical form by means of a similarity transformation:

$$X(0) = T\Lambda_0 T^{-1}, \;\; TT^{-1} = I_n, \tag{S14}$$

with $\Lambda_0 = \text{diag}(A_1, \ldots, A_k, B_1, \ldots, B_l)$, where A_i are real Jordan blocks and

$$B_j = \begin{pmatrix} C_i & I_2 & \ldots & 0 \\ 0 & C_i & \ddots & 0 \\ \vdots & \vdots & \ddots & \vdots \\ 0 & 0 & \ldots & C_i \end{pmatrix}, C_j = \alpha_j I_2 + \beta_j J_2, \tag{S15}$$

with $\beta_j \neq 0$.

We again observe that if $\Lambda(t)$ is the solution to the initial value problem $\dot{\Lambda} = \Lambda^2$, $\Lambda(0) = \Lambda_0$, then $X(t) := T\Lambda(t)T^{-1}$, is the solution to Eq. S5. Again, it is sufficient to solve system S5 in case of specific block-triangular X of the form A_i or B_j as in S15. To deal with the first form A_i, we first we consider more general, triangular Toeplitz initial conditions:

$$X(0) = \begin{pmatrix} x_1(0) & x_2(0) & \cdots & x_n(0) \\ 0 & x_1(0) & \ddots & x_{n-1}(0) \\ \vdots & \vdots & \ddots & \vdots \\ 0 & 0 & \cdots & x_1(0) \end{pmatrix}, \tag{S16}$$

with $x_i(0)$ reals, and denote $\mathcal{TT} = \{X \mid X \text{ is of the form } S16\}$. It turns out that this is an invariant set for the system, which can be easily verified by noting that if X belongs to $\mathcal{TT}$, then so does X^2.

Lemma 2. *Let $X(0) \in \mathcal{TT}$ with*

$$x_i(0) = \begin{cases} a \neq 0 \;\; \textit{if} \; i = 1 \\ 1 \;\; \textit{if} \; i = 2 \\ 0 \;\; \textit{otherwise} \end{cases}.$$

Then the forward solution $X(t)$ of S5 is defined on $[0, t^*)$ where $t^* = 1/a$ if $a > 0$ and on $t^* = \infty$ if $a \leq 0$, belongs to $\mathcal{TT}$, and satisfies

$$x_i(t) = p_i\left(\frac{1}{1 - at}\right), \;\; t \in [0, t^*),$$

where each $p_i(z)$ is a polynomial of degree i:

$$p_i(z) = \begin{cases} az \;\; \textit{if} \; i = 1 \\ \frac{1}{a^{i-2}} z^i + \cdots + c_i z^2 \;\; \textit{otherwise} \end{cases}, \tag{S17}$$

where c_i is some real constant, so that $p_i(z)$ has no constant or first order terms when $i > 1$.

Proof. First note that system S5 can be solved recursively, starting with $x_1(t)$, followed by $x_2(t), x_3(t), \dots$. Only the first equation for x_1 is nonlinear, whereas the equations for $x_2, x_3, \dots$ are linear. To see this, we write these equations:

$$\dot{x}_i = \begin{cases} x_1^2, \;\; x_1(0) = a \text{ if } i = 1 \\ (2x_1(t))x_2, \;\; x_2(0) = 1 \text{ if } i = 2 \\ (2x_1(t))x_i + \sum_{k=2}^{i-1} x_k(t) x_{i-(k-1)}(t), \;\; x_i(0) = 0 \text{ if } i > 2 \end{cases}.$$

The forward solution for x_1 is $x_1(t) = \frac{a}{1-at}$, for $t \in [0, t^*)$, which establishes the result if $i = 1$. The forward solution for x_2 is: $x_2(t) = \frac{1}{(1-at)^2}$, for $t \in [0, t^*)$, which establishes the result if $i = 2$. If $i > 2$, we obtain the proof by induction on n. Assume the result holds for $i = 1, \dots, n$, for some $n \geq 2$, and consider the equation for x_{n+1}. Using that $x_n(0) = 0$ for $n \geq 2$, the solution is given by:

$$x_{n+1}(t) = \mathrm{e}^{\int_0^t 2x_1(s)ds} \left[0 + \int_0^t \left(\sum_{k=2}^{n} x_k(s) x_{n-k+2}(s) \right) \mathrm{e}^{\int_0^s -2x_1(\tau)d\tau} \, ds \right].$$

Since $\mathrm{e}^{\int_0^t 2x_1(s)ds} = x_2(t)$ and thus $\mathrm{e}^{\int_0^s -2x_1(\tau)d\tau} = 1/x_2(s)$, it follows that:

$$x_{n+1}(t) = \frac{1}{(1-at)^2} \left[\int_0^t \Big(\sum_{k=2}^{n} p_k(1/(1-as)) p_{n-k+2}(1/(1-as)) \Big) (1-as)^2 ds \right].$$

Since the polynomials appearing in the integral take the form of Eq. $S17$, they are all missing first order and constant terms, and thus there follows that

$$x_{n+1}(t) = \frac{1}{(1-at)^2} \left[\int_0^t \Big(\sum_{k=2}^{n} \frac{1}{a^{n-2}} \frac{1}{(1-as)^{n+2}} + \dots + c_k c_{n-k+2} \frac{1}{(1-as)^4} \Big) (1-as)^2 ds \right]$$

and so that

$$x_{n+1}(t) = \frac{1}{a^{n-1}} \frac{1}{(1-at)^{n+1}} + \dots + \frac{c_{n+1}}{(1-at)^2}, \;\; t \in [0, t^*),$$

where K_{n+1} and c_{n+1} are certain constants (which are related in some way which is irrelevant for what follows). This shows that $x_{n+1}(t)$ is indeed of the form $p_{n+1}(1/(1-at))$ with $p_{n+1}(z)$ as in S17. □

Next we consider equation S5 in case $X(0)$ is a block triangular Toeplitz initial condition:

$$X(0) = \begin{pmatrix} B_1(0) & B_2(0) & \cdots & B_n(0) \\ 0 & B_1(0) & \ddots & B_{n-1}(0) \\ \vdots & \vdots & \ddots & \vdots \\ 0 & 0 & \cdots & B_1(0) \end{pmatrix}, \tag{S18}$$

with $B_i(0) = \alpha_i I_2 + \beta_i J_2$ with $\alpha_i, \beta_i \in \mathbb{R}$, and denote $\mathcal{BTT} = \{X \mid X \text{ is of the form } S18\}$. Again the set $\mathcal{BTT}$ is invariant for system S5. We use this to solve equation S5 in case $X(0)$ is a real Jordan block corresponding to a pair of eigenvalues $\alpha \pm j\beta$.

Lemma 3. *Let $X(0) \in \mathcal{BTT}$ with*

$$B_i(0) = \begin{cases} \alpha I_2 + \beta J_2 \;\; \textit{if } i = 1 \\ I_2 \;\; \textit{if } i = 2 \\ 0 \;\; \textit{otherwise} \end{cases}.$$

Then the forward solution $X(t)$ of S5 is defined on $[0, +\infty)$, and it belongs to $\mathcal{BTT}$.

Proof. Just like in the proof of Proposition 2, we note that system S5 can be solved recursively, starting with $X_1(t)$, followed by $X_2(t), X_3(t), \ldots$. Only the first equation for X_1 is nonlinear, whereas the equations for $X_2, X_3, \ldots$ are linear. To see this, we write these equations:

$$\dot{X}_i = \begin{cases} X_1^2, \ \ X_1(0) = \alpha I_2 + \beta J_2 \text{ if } i = 1 \\ (2X_1(t))X_2, \ \ X_2(0) = I_2 \text{ if } i = 2 \\ (2X_1(t))X_i + \sum_{k=2}^{i-1} X_k(t)X_{i-(k-1)}(t), X_i(0) = 0 \text{ if } i > 2 \end{cases}.$$

Here we have used the fact that $X_1X_i + X_iX_1 = 2X_1X_i$, since any two matrices of the form $pI_2 + qJ_2$ commute and the matrices $X_i(t)$ are of this form.

By Lemma 1, the forward solution for $X_1(t)$ is defined for all t in $[0, +\infty)$ (and in fact, converges to zero as $t \to +\infty$).

Since the $X_1(t)$ commute for every pair of t's, the forward solution for $X_2(t)$ is given by [6] $X_2(t) = e^{\int_0^t 2X_1(s)ds}$, for $t \in [0, +\infty)$, where this solution exists for all forward times t because $X_1(t)$ is bounded and continuous. Similarly, the forward solution for $X_i(t)$ when $i > 2$, is given by the variation of constants formula:

$$X_i(t) = X_2(t)\left[\int_0^t X_2^{-1}(s)\left(\sum_{k=2}^{i-1} X_k(s)X_{i-(k-1)}(s)\right) ds\right],$$

for $t \in [0, +\infty)$ when $i > 2$, where these solutions are recursively defined for all forward times because the formula only involves integrals of continuous functions. □

Combining both results, puts us in a position to state and prove our main result.

Theorem 3. *Let $X(0) \in \mathbb{R}^{n\times n}$ and (T, Λ_0) as in S14 with S15. Let $a_1 > a_2 \geq \cdots \geq a_k$ with $a_1 > 0$ a simple eigenvalue with corresponding right and left-eigenvectors U_1 and V_1^T respectively:*

$$X(0)U_1 = a_1U_1 \text{ and } V_1^TX(0) = a_1V_1^T.$$

Then the forward solution $X(t)$ of S5 is defined for $[0, 1/a_1)$, and

$$\lim_{t\to 1/a_1} \frac{X(t)}{|X(t)|_F} = \frac{U_1V_1^T}{|U_1V_1^T|_F}.$$

Proof. Consider the initial value problem $\dot{\Lambda} = \Lambda^2, \ \Lambda(0) = \Lambda_0$, whose solution is given by

$$\Lambda(t) = \text{diag}(A_1(t), \ldots, A_k(t), B_1(t), \ldots, B_l(t)),$$

where for all $i = 1, \ldots, k$, $A_i(t)$ is the forward solution of S5 with $A_i(0)$ of the form A_i in S15, which by Lemma 2 is defined for all $t \in [0, 1/a_i)$. Since $a_1 > a_2 \geq \cdots \geq a_k$, $A_1(t)$ blows up first when $t \to 1/a_1$. The matrices $B_j(t)$, $j = 1, \ldots, l$, are the forward solution of S5 with $B_j(0)$ of the form B_j in S15, and by Lemma 3, they are defined for all t in $[0, +\infty)$.

This clearly shows that $\Lambda(t)$ is defined in forward time for t in $[0, 1/a_1)$. Since the solution of S5 is given by $X(t) = T\Lambda(t)T^{-1}$, $X(t)$ is also defined in forward time for t in $[0, 1/a_1)$, and it follows that

$$\lim_{t\to 1/a_1} \frac{X(t)}{|X(t)|_F} = \lim_{t\to 1/a_1} \frac{T\Lambda(t)T^{-1}}{|X(t)|_F} = \frac{Te_1e_1^TT^{-1}}{|Te_1e_1^TT^{-1}|_F} = \frac{U_1V_1^T}{|U_1V_1^T|_F},$$

where e_1 denotes the first standard unit basis vector of $\mathbb{R}^n$. □

Theorem 3 implies that social balance is usually not achieved when $X(0)$ is an arbitrary real initial condition. Indeed, if X_0 has a simple, positive, real eigenvalue a_1, and if we assume that no entry of the right and left eigenvectors U_1 and V_1^T are zero (an assumption which is generically satisfied), then in general, up to a permutation of its entries, the sign patterns of U_1 and V_1^T are:

$$U_1 = \begin{pmatrix} + \\ + \\ \hline - \\ - \end{pmatrix} \text{ and } V_1^T = \begin{pmatrix} + & - & | & + & - \end{pmatrix}$$

implies that

$$U_1 V_1^T = \left(\begin{array}{cc|cc} + & - & + & - \\ + & - & + & - \\ \hline - & + & - & + \\ - & + & - & + \end{array}\right).$$

Then Theorem 1 implies that the normalized state of the system does not become balanced in finite time.

This shows that in general, unless X_0 is normal (so that Theorem 2 is applicable), we cannot expect that social balance will emerge for system S5.

Equation $\dot{X} = XX^T$

We now consider

$$\dot{X} = XX^T, X(0) = X_0, \tag{S19}$$

where again, each X_{ij} denotes the real-valued opinion agent i has about agent j. As before, for $i = j$, the value of X_{ii} is interpreted as a measure of self-esteem of agent i. We can also write the equations entrywise:

$$\dot{X}_{ij} = \sum_k X_{ik} X_{jk}. \tag{S20}$$

As in the case of model $\dot{X} = X^2$, we split up the analysis in two parts. First we consider system S19 with normal initial condition X_0, and we shall see that not all initial conditions lead to the emergence of a balanced network in this case, in contrast to the behaviour of S5. Secondly, we will see that for non-normal, generic initial conditions X_0, we typically do get the emergence of social balance, also contrasting the behaviour of S5.

Normal initial condition

As for the model $\dot{X} = X^2$ the set $\mathcal{N}$ is invariant for system S19. By using the same diagonalisation as in Eq. S7, if $\Lambda(t)$ is the solution to the initial value problem $\dot{\Lambda} = \Lambda\Lambda^T$, $\Lambda(0) = \Lambda_0$, then $X(t) := U\Lambda(t)U^T$, is the solution to Eq. S19. This shows it is sufficient to solve system S19 in case of scalar X or in case of a specific 2×2 normal matrix X. The scalar case is easy to solve and follows Eq. S8, so we turn to the 2×2 case by considering

$$\dot{X} = XX^T, \; X(0) = \alpha I_2 + \beta J_2, \text{ where } \beta \neq 0. \tag{S21}$$

We define the angle ϕ as

$$\phi = \arctan\left(\frac{\alpha}{\beta}\right), \; \phi \in \left(-\frac{\pi}{2}, \frac{\pi}{2}\right). \tag{S22}$$

Lemma 4. *Define $\bar{t}$ as*

$$\bar{t} = \frac{\pi}{2\beta} - \frac{\phi}{\beta}. \tag{S23}$$

Then the forward solution $X(t)$ of S21 is:

$$X(t) = \beta \tan(\beta t + \phi) I_2 + \beta J_2, \ \ t \in [0, \bar{t}). \tag{S24}$$

Moreover,

$$\lim_{t \to \bar{t}-} X(t) = +\infty I_2 + \beta J_2 \ \text{and} \ \lim_{t \to \bar{t}-} \frac{X(t)}{|X(t)|_F} = \frac{\sqrt{2}}{2} I_2.$$

Proof. Let $X_0 = S_0 + A_0$, $S_0 = \alpha I_2$, and $A_0 = \beta J_2$. Then the solution $X(t)$ of S21 can be decomposed as $S(t) + A(t)$, where

$$\dot{S} = (S + A)(S - A), \ \ S(0) = S_0, \tag{S25}$$

$$\dot{A} = 0, \ \ A(0) = A_0, \tag{S26}$$

so $A(t) = A_0$, and reduces to

$$\dot{S} = (S + A_0)(S - A_0), \ \ S(0) = S_0 \tag{S27}$$

Note that S27 is a matrix Riccati differential equation with the property that the line $\mathcal{L} = \{\alpha I_2 | \alpha \in \mathbb{R}\}$, is an invariant set under the flow. Therefore it suffices to solve the scalar Riccati differential equation corresponding to the dynamics of the diagonal entries of S: $\dot{s} = s^2 + \beta^2$, $s(0) = \alpha$, whose forward solution is: $s(t) = \beta \tan(\beta t + \phi)$, for $t \in (0, \bar{t})$, where $\bar{t}$ is given by S23. Consequently, the forward solution $X(t)$ of S21 is given by: $X(t) = S(t) + A_0 = \beta \tan(\beta t + \phi) I_2 + \beta J_2$, for $t \in (0, \bar{t})$, and thus $\lim_{t \to \bar{t}-} X(t) = +\infty I_2 + \beta J_2$ and

$$\lim_{t \to \bar{t}-} \frac{X(t)}{|X(t)|_F} = \frac{X(t)}{\sqrt{2} |\beta \sec(\beta t + \phi)|} = \frac{\sqrt{2}}{2} I_2.$$

□

Combining the solution for the 1×1 scalar case in Eq. S8 and Lemma 4 yields our main result:

Theorem 4. *Let $X_0 \in \mathcal{N}$, and let (U, Λ_0) be as in Lemma S7. Define*

$$\bar{t}_i = \begin{cases} 1/a_i & \text{if } a_i > 0 \\ +\infty & \text{if } a_i \leq 0 \end{cases} \quad \text{for all } i = 1, \ldots, k,$$

and

$$\bar{t}_j = \frac{\pi}{2\beta_j} - \frac{\phi_j}{\beta_j} \ \text{for all } j = 1, \ldots, l,$$

where $\phi_j = \arctan\left(\frac{\alpha_j}{\beta_j}\right)$ and let $\bar{t} = \min_{i,j}\{\bar{t}_i, \bar{t}_j\}$. Then the forward solution $X(t)$ of S19 is defined for $[0, \bar{t})$.

If there is a unique $i^* \in \{1, \ldots, k\}$ such that $\bar{t} = \bar{t}_{i^*}$ is finite, then

$$\lim_{t \to \bar{t}_{i^*}-} \frac{X(t)}{|X(t)|_F} = U_{i^*} U_{i^*}^T,$$

where U_{i^*} is the i^*th column of U, an eigenvector corresponding to eigenvalue a_{i^*} of X_0.

If there is a unique $j^* \in \{1, \dots, l\}$ *such that* $\bar{t} = \bar{t}_{j^*}$*, then*

$$\lim_{t \to \bar{t}_{j^*}-} \frac{X(t)}{|X(t)|_F} = \frac{\sqrt{2}}{2} U_{j^*} U_{j^*}^T,$$

where U_{j^*} *is an* $n \times 2$ *matrix consisting of the two consecutive columns of* U *which correspond to the columns of the* 2×2 *block* B_{j^*} *in* Λ_0*.*

Proof. Consider the initial value problem:

$$\dot{\Lambda} = \Lambda \Lambda^T, \ \Lambda(0) = \Lambda_0.$$

By Lemma 4 its solution is given by

$$\Lambda(t) = \begin{pmatrix} \frac{a_1}{1-a_1 t} & \dots & 0 & 0 & \dots & 0 \\ \vdots & \ddots & \vdots & \vdots & \ddots & \vdots \\ 0 & \dots & \frac{a_k}{1-a_k t} & 0 & \dots & 0 \\ 0 & \dots & 0 & X_1(t) & \dots & 0 \\ \vdots & \ddots & \vdots & \vdots & \ddots & \vdots \\ 0 & \dots & 0 & 0 & \dots & X_l(t) \end{pmatrix},$$

where for all $j = 1, \dots, l$, $X_j(t)$ is given by the 2×2 matrix in S24 with β, ϕ and $\bar{t}$ replaced by β_j, ϕ_j and $\bar{t}_j$ respectively. This clearly shows that $\Lambda(t)$ is defined in forward time for t in $[0, \bar{t})$. Since the solution of S19 is given by $X(t) = U\Lambda(t)U^T$, $X(t)$ is also defined in forward time for t in $[0, \bar{t})$. It follows from S2 that

$$\frac{X(t)}{|X(t)|_F} = U \frac{\Lambda(t)}{|\Lambda(t)|_F} U^T.$$

If $i^* \in \{1, \dots, k\}$ is the unique value such that $\bar{t} = \bar{t}_{i^*}$, then

$$\begin{aligned} \lim_{t \to \bar{t}_i^*} \frac{X(t)}{|X(t)|_F} &= U \lim_{t \to \bar{t}_i^*} \frac{\Lambda(t)}{|\Lambda(t)|_F} U^T \\ &= U e_{i^*} e_{i^*}^T U^T = U_{i^*} U_{i^*}^T, \end{aligned}$$

where e_{i^*} denotes the i^*th standard unit basis vector of $\mathbb{R}^n$.

If $j^* \in \{1, \dots, l\}$ is the unique value such that $\bar{t} = \bar{t}_{j^*}$, then by Lemma 4:

$$\begin{aligned} \lim_{t \to \bar{t}_j^*} \frac{X(t)}{|X(t)|_F} &= U \lim_{t \to \bar{t}_j^*} \frac{\Lambda(t)}{|\Lambda(t)|_F} U^T \\ &= \frac{\sqrt{2}}{2} U E_{j^*} U^T = \frac{\sqrt{2}}{2} U_{j^*} U_{j^*}^T, \end{aligned}$$

where E_{j^*} has exactly two non-zero entries equal to 1 on the diagonal positions corresponding to the block B_{j^*} in Λ_0. □

A particular consequence of Theorem 4 is that if X_0 has a complex pair of eigenvalues, the solution of S19 always blows up in finite time, even if all real eigenvalues of X_0 are non-positive. Recall that the solution of S5 blows up in finite time, if and only if X_0 has a positive, real eigenvalue. Another implication of Theorem 4 is that if blow-up occurs, it may be due to a real eigenvalue of X_0, or to a complex eigenvalue. In contrast, if the solution of S5 blows up in finite time, it is necessarily due to a positive, real eigenvalue, and never to a complex eigenvalue. When the solution of S19 blows up because

of a positive, real eigenvalue of X_0, the system will achieve balance, just as in the case of system S5. If on the other hand, finite time blow up of S19 is caused by a complex eigenvalue of X_0, we show that in general one cannot expect to achieve a balanced network. Assume there is a unique j^* such that:

$$\lim_{t\to \bar{t}_j^*-} \frac{X(t)}{|X(t)|_F} = \frac{\sqrt{2}}{2} U_{j^*} U_{j^*}^T,$$

Assuming that no entry of U_{j^*} is zero, the sign pattern of $U_{j^*} U_{j^*}^T$, with

$$U_j^* = \begin{pmatrix} p_1 & q_1 \\ p_2 & -q_2 \\ -p_3 & q_3 \\ -p_4 & -q_4 \end{pmatrix}$$

is given by:

$$\begin{pmatrix} + & ? & ? & - \\ ? & + & - & ? \\ ? & - & + & ? \\ - & ? & ? & + \end{pmatrix},$$

up to a suitable permutation, where all p_i and q_i, $i = 1, \dots, 4$, are entrywise positive vectors, and where

$$\langle p_1, q_1 \rangle + \langle p_4, q_4 \rangle = \langle p_2, q_2 \rangle + \langle p_3, q_3 \rangle,$$

because U is an orthogonal matrix. The ? are not entirely arbitrary because $U_{j^*} U_{j^*}^T$ is a symmetric matrix, but besides that their signs can be arbitrary.

Generic initial condition

Consider

$$\dot{X} = XX^T, X(0) = X_0, \tag{S28}$$

where X is a real $n \times n$ matrix, which is not necessarily normal.

We first decompose the flow S28 into flows for the symmetric and skew-symmetric parts of X. Let $X = S + A$,$X_0 = S_0 + A_0$, where $S, S_0 \in \mathcal{S}$ and $A, A_0 \in \mathcal{A}$ are the unique symmetric and skew-symmetric parts of X and X_0 respectively. If $X(t)$ satisfies S28, then it can be verified that $S(t)$ and $A(t)$ satisfy the system:

$$\dot{S} = (S + A)(S - A),\ S(0) = 0, \tag{S29}$$

$$\dot{A} = 0,\ A(0) = A_0, \tag{S30}$$

Consequently, $A(t) = A_0$ for all t, and thus the skew-symmetric part of the solution $X(t)$ of S28 remains constant and equal to A_0. Throughout this subsection we assume that $A_0 \neq 0$, for otherwise $X(0)$ is symmetric, hence normal, and the results from the previous subsection apply. It follows that we only need to understand the dynamics of the symmetric part. Then the solution $X(t)$ to S28 is given by $X(t) = S(t) + A_0$, where $S(t)$ solves S29, and in view of S1, there follows by Pythagoras' Theorem that: $|X(t)|_F^2 = |S(t)|_F^2 + |A_0|_F^2$, and thus

$$\frac{X(t)}{|X(t)|_F} = \frac{S(t) + A_0}{(|S(t)|_F^2 + |A_0|_F^2)^{\frac{1}{2}}}. \tag{S31}$$

Next we shall derive an explicit expression for the solution $S(t)$ of S29. We start by performing a change of variables:

$$\hat{S}(t) = \mathrm{e}^{-tA_0} S(t) \, \mathrm{e}^{tA_0}. \tag{S32}$$

This yields the equation

$$\dot{\hat{S}} = \hat{S}^2 - A_0^2, \;\; \hat{S}(0) = S_0. \tag{S33}$$

We perform a further transformation which diagonalizes $-A_0^2$: Let V be an orthogonal matrix such that $-V^T A_0^2 V = D^2$, where $D := \text{diag}(0, \omega_1 I_2, \ldots, \omega_k I_k)$ where $k \geq 1$ (because $A_0 \neq 0$) and all $\omega_j > 0$ without loss of generality. Setting

$$\tilde{S} = V^T \hat{S} V, \tag{S34}$$

and multiplying equation S33 by V on the left, and by V^T on the right, we find that:

$$\dot{\tilde{S}} = \tilde{S}^2 + D^2, \;\; \tilde{S}(0) = \tilde{S}_0 := V^T S_0 V. \tag{S35}$$

Notice that this is a matrix Riccati differential equation, a class of equations with specific properties which are briefly reviewed next.

Consider a general matrix Riccati differential equation:

$$\dot{S} = SMS - SL - L^T S + N, \tag{S36}$$

where $M = M^T$,$N = N^T$ and L arbitrary, defined on $\mathcal{S}$. Associated to this equation is a linear system

$$\begin{pmatrix} \dot{P} \\ \dot{Q} \end{pmatrix} = H \begin{pmatrix} P \\ Q \end{pmatrix}, \;\; H := \begin{pmatrix} L & -M \\ N & -L^T \end{pmatrix}, \tag{S37}$$

where H is a Hamiltonian matrix, i.e. $J_{2n} H = (J_{2n} H)^T$ holds, where J_{2n} is as defined in Eq. S3. The following fact is well-known.

Lemma 5. *Let $\begin{pmatrix} P(t) \\ Q(t) \end{pmatrix}$ be a solution of S37. Then, provided that $P(t)$ is non-singular,*

$$S(t) = Q(t) P(t)^{-1}, \tag{S38}$$

is a solution of S36. Conversely, if $S(t)$ is a solution of S36, then there exists a solution $\begin{pmatrix} P(t) \\ Q(t) \end{pmatrix}$ of S37 such that S38 holds, provided that $P(t)$ is non-singular.

Proof. Taking derivatives in $S(t)P(t) = Q(t)$ yields that $\dot{S} = (\dot{Q} - S\dot{P})P^{-1}$, and using S37,

$$\dot{S} = (NP - L^T Q - S(LP - MQ))P^{-1} = N - L^T S - SL + SMS,$$

showing that $S(t)$ solves S36. For the converse, let $S(t)$ be a solution of S36. Let $\begin{pmatrix} P(t) \\ Q(t) \end{pmatrix}$ with $\begin{pmatrix} P(0) \\ Q(0) \end{pmatrix} = \begin{pmatrix} I_n \\ S(0) \end{pmatrix}$ be the solution of S37. Then

$$\begin{aligned} &\frac{d}{dt}\left(Q(t)P^{-1}(t)\right) \\ =&\dot{Q}P^{-1} - QP^{-1}\dot{P}P^{-1} \\ =&(NP - L^T Q)P^{-1} - QP^{-1}(LP - MQ)P^{-1} \\ =&(QP^{-1})M(QP^{-1}) - (QP^{-1})L - L^T(QP^{-1}) + N, \end{aligned}$$

which implies that QP^{-1} is a solution to S36. Since $S(0) = Q(0)P^{-1}(0)$, it follows from uniqueness of solutions that $S(t) = Q(t)P^{-1}(t)$. □

In other words, in principle we can solve the nonlinear equation S36 by first solving the linear system S37, and then use formula S38 to determine the solution of S36.

We carry this out for our particular Riccati equation S35 which is of the form S36 if $M = I_n$, $L = 0$, $N = D^2$. The corresponding Hamiltonian is $H = \begin{pmatrix} 0 & -I_n \\ D^2 & 0 \end{pmatrix}$. We partition D in singular and non-singular parts:

$$D = \begin{pmatrix} 0 & 0 \\ 0 & \tilde{D} \end{pmatrix}, \text{ where } \tilde{D} := \begin{pmatrix} \omega_1 I_2 & \dots & 0 \\ \vdots & \ddots & \vdots \\ 0 & \dots & \omega_k I_2 \end{pmatrix},$$

where $\tilde{D}$ is positive definite since all $\omega_j > 0$. Partitioning H correspondingly:

$$H = \left(\begin{array}{cc|cc} 0 & 0 & -I_{n-2k} & 0 \\ 0 & 0 & 0 & -I_{2k} \\ \hline 0 & 0 & 0 & 0 \\ 0 & \tilde{D}^2 & 0 & 0 \end{array}\right). \tag{S39}$$

This matrix is then exponentiated to solve system S37:

$$\begin{pmatrix} P(t) \\ Q(t) \end{pmatrix} = \left(\begin{array}{cc|cc} I_{n-2k} & 0 & -tI_{n-2k} & 0 \\ 0 & c & 0 & -\tilde{D}^{-1}s \\ \hline 0 & 0 & I_{n-2k} & 0 \\ 0 & \tilde{D}s & 0 & c \end{array}\right) \begin{pmatrix} P(0) \\ Q(0) \end{pmatrix},$$

where we have introduced the following notation:

$$s(t) := \operatorname{diag}(\sin(\omega_1 t)I_2, \dots, \sin(\omega_k t)I_2) = \sin(\tilde{D}t),$$

and similarly $c(t) = \cos(\tilde{D}t)$. By setting $P(0) = I_n$, and $Q(0) = \tilde{S}_0$, and using Lemma 5, it follows that the solution of the initial value problem S35 is given by $\tilde{S}(t) = Q(t)P(t)^{-1}$,

$$\begin{pmatrix} P(t) \\ Q(t) \end{pmatrix} = \left(\begin{array}{c} \begin{pmatrix} (I_{n-2k} - t)\tilde{S}_0 & 0 \\ 0 & c(t) - \tilde{D}^{-1}s(t)\tilde{S}_0 \end{pmatrix} \\ \hline \begin{pmatrix} I_{n-2k}\tilde{S}_0 & 0 \\ 0 & \tilde{D}s(t) + c(t)\tilde{S}_0 \end{pmatrix} \end{array}\right), \tag{S40}$$

for all t for which $P(t)$ is non-singular. We now make the following assumption:

Assumption A. *The matrix $P(t)$ is non-singular for all t in $[0, \bar{t})$, where $\bar{t}$ is finite and such that $s(t)$ is non-singular for all t in $(0, \bar{t})$. Moreover, $P(\bar{t})$ has rank $n-1$, or equivalently, has a simple eigenvalue at zero.*

Later we will show that this assumption is generically satisfied, and also that

$$t^* = \bar{t}, \tag{S41}$$

where $[0, t^*)$ is the maximal forward interval of existence of the solution $\tilde{S}(t)$ of the initial value problem S35. Consequently, the theory of ODE's implies that $\lim_{t\to\bar{t}} |\tilde{S}(t)|_F = +\infty$, i.e. that $\bar{t}$ is the blow-up time for the solution $\tilde{S}(t)$.

Assuming for the moment that assumption A is satisfied, back-transformation using S32 and S34, yields that the solution $S(t)$ of S29 is $S(t) = \mathrm{e}^{tA_0} V\tilde{S}(t)V^T \mathrm{e}^{-tA_0}$, which is defined for all t in $[0, \bar{t})$, because $\mathrm{e}^{tA_0} V$ is bounded for all t (as it is an orthogonal matrix). It follows from S2 that

$$\lim_{t\to\bar{t}} \frac{S(t)}{|S(t)|_F} = \mathrm{e}^{\bar{t}A_0} V \left(\lim_{t\to\bar{t}} \frac{\tilde{S}(t)}{|\tilde{S}(t)|_F} \right) V^T \mathrm{e}^{-\bar{t}A_0}, \tag{S42}$$

provided that at least one of the two limits exists. Partitioning $\tilde{S}_0$ in S40 as follows:

$$\tilde{S}_0 = \begin{pmatrix} (\tilde{S}_0)_{11} & (\tilde{S}_0)_{12} \\ (\tilde{S}_0)_{12}^T & (\tilde{S}_0)_{22} \end{pmatrix}, \text{ with } \begin{array}{c} (\tilde{S}_0)_{11} = (\tilde{S}_0)_{11}^T \\ (\tilde{S}_0)_{22} = (\tilde{S}_0)_{22}^T \end{array},$$

we can rewrite $P(t)$ and $Q(t)$ on the time interval $(0,\bar{t})$ as: $P(t) = \Delta(t)M(t)$ with,

$$\Delta(t) = \begin{pmatrix} tI_{n-2k} & 0 \\ 0 & \tilde{D}^{-1}s(t) \end{pmatrix},$$

and

$$M(t) = \begin{pmatrix} 1/t - (\tilde{S}_0)_{11} & -(\tilde{S}_0)_{12} \\ -(\tilde{S}_0)_{12}^T & \tilde{D}c(t)s^{-1}(t) - (\tilde{S}_0)_{22} \end{pmatrix} = M^T(t),$$

and

$$Q(t) = \begin{pmatrix} (\tilde{S}_0)_{11} & (\tilde{S}_0)_{12} \\ c(t)(\tilde{S}_0)_{12}^T & \tilde{D}s(t) + c(t)(\tilde{S}_0)_{22} \end{pmatrix}.$$

Note that the factorisation of $P(t)$ is well-defined on $(0,\bar{t})$ because by assumption A, the matrix $s(t)$ is non-singular in the interval $(0,\bar{t})$. Moreover, assumption A also implies there exists a nonzero vector u corresponding to the zero eigenvalue of $M(\bar{t})$, i.e. $M(\bar{t})u = 0$, and that u is uniquely defined up to scalar multiplication because the zero eigenvalue is simple. More explicitly, partitioning u as $\left(\begin{smallmatrix} u_1 \\ u_2 \end{smallmatrix}\right)$, there holds that

$$\begin{pmatrix} 1/\bar{t} - (\tilde{S}_0)_{11} & -(\tilde{S}_0)_{12} \\ -(\tilde{S}_0)_{12}^T & \tilde{D}c(\bar{t})s^{-1}(\bar{t}) - (\tilde{S}_0)_{22} \end{pmatrix} \begin{pmatrix} u_1 \\ u_2 \end{pmatrix} = 0. \tag{S43}$$

Notice that $M(t)$ is at least real-analytic on the interval $(0,\bar{t})$. Hence, it follows from [7] (see also [8,9]), that there is an orthogonal matrix $U(t)$, and a diagonal matrix $\Lambda(t)$, both real-analytic on $(0,\bar{t})$, such that: $M(t) = U(t)\Lambda(t)U^T(t)$, for $t \in (0,\bar{t})$, and thus $M^{-1}(t) = U(t)\Lambda^{-1}(t)U^T(t)$, for $t \in (0,\bar{t})$. Returning to S42, we obtain that:

$$\lim_{t\to\bar{t}} \frac{S(t)}{|S(t)|_F} = \mathrm{e}^{\bar{t}A_0} V \lim_{t\to\bar{t}} \frac{Q(t)U(t)\Lambda^{-1}(t)U^T(t)\Delta^{-1}(t)}{|Q(t)U(t)\Lambda^{-1}(t)U^T(t)\Delta^{-1}(t)|_F} V^T \mathrm{e}^{-\bar{t}A_0} = \mathrm{e}^{\bar{t}A_0} V \frac{Q(\bar{t})uu^T\Delta^{-1}(t)}{|Q(\bar{t})uu^T\Delta^{-1}(t)|_F} V^T \mathrm{e}^{-\bar{t}A_0}.$$

Here, we have used the fact that $M^{-1}(t)$ is positive definite on the interval $(0,\bar{t})$, so that its largest eigenvalue (which is simple for all $t < \bar{t}$ sufficiently close to $\bar{t}$, because of assumption A approaches $+\infty$ -and not $-\infty$- as $t \to \bar{t}$. To see this, note that from its definition follows that $M(t)$ is positive definite for all sufficiently small $t > 0$, because $\tilde{D}$ is positive definite. Moreover, $M(t)$ is non-singular on $(0,\bar{t})$ since by assumption (**A**), $P(t)$ is non-singular on $(0,\bar{t})$, and because $M(t) = \Delta^{-1}(t)P(t)$ (it is clear from its definition and assumption A that $\Delta(t)$ is non-singular on $(0,\bar{t})$ as well). Consequently, the smallest eigenvalue of $M(t)$ remains positive in $(0,\bar{t})$, as it approaches zero as $t \to \bar{t}$. This implies that the largest eigenvalue of $M^{-1}(t)$ is positive on $(0,\bar{t})$, and approaches $+\infty$ as $t \to \bar{t}$, as claimed.

Note that:

$$\begin{aligned} Q(\bar{t})u &= \begin{pmatrix} (\tilde{S}_0)_{11} & (\tilde{S}_0)_{12} \\ c(\bar{t})(\tilde{S}_0)_{12}^T & \tilde{D}s(\bar{t}) + c(\bar{t})(\tilde{S}_0)_{22} \end{pmatrix} \begin{pmatrix} u_1 \\ u_2 \end{pmatrix} \\ &= \begin{pmatrix} (1/\bar{t})u_1 \\ \tilde{D}s^{-1}(\bar{t})u_2 \end{pmatrix} = \Delta^{-1}(\bar{t})u, \end{aligned}$$

where in the second equality, we used the second row of S43 , multiplied by $c(\bar{t})$. From this follows that

$$\lim_{t\to\bar{t}} \frac{S(t)}{|S(t)|_F} = \mathrm{e}^{\bar{t}A_0} V \frac{\Delta^{-1}(\bar{t})uu^T\Delta^{-1}(\bar{t})}{|\Delta^{-1}(\bar{t})uu^T\Delta^{-1}(\bar{t})|_F} V^T \mathrm{e}^{-\bar{t}A_0} = \frac{ww^T}{|ww^T|_F},$$

where $w := e^{\bar{t}A_0} V \Delta^{-1}(\bar{t}) u$.

Taking limits for $t \to \bar{t}$ in S31, and using the above equality, we finally arrive at the following result, which implies that system S28 evolves to a socially balanced state (in normalized sense) when $t \to \bar{t}$:

Proposition 1. *Suppose that assumption A holds and $A_0 \neq 0$. Then the solution $X(t)$ of S28 satisfies:*

$$\lim_{t \to \bar{t}} \frac{X(t)}{|X(t)|_F} = \frac{ww^T}{|ww^T|_F}.$$

with $w = e^{\bar{t}A_0} V \Delta^{-1}(\bar{t}) u$.

Genericity

Generically, assumption A holds, and S41 holds as well. There are two aspects to assumption A:

1. The matrix $P(t)$ is nonsingular in the interval $[0, \bar{t})$, but singular at some finite $\bar{t}$ such that:

$$\bar{t} < \min_{j=1,\ldots,k} \frac{\pi}{\omega_j}. \tag{S44}$$

2. $P(\bar{t})$ has a simple zero eigenvalue.

To deal with the first item, suppose that the solution $\tilde{S}(t)$ of S35 is defined for all $t \in [0, t^*)$ for some finite positive t^*. By Lemma 5, there exist $P(t)$ and $Q(t)$ such that $\tilde{S}(t) = Q(t)P^{-1}(t)$, where $P(t)$ and $Q(t)$ are components of the solution of system S37 with H defined in S39. Then necessarily $\bar{t} \leq t^*$. Thus, if we can show that $t^* < \min_j \pi/\omega_j$, then S44 holds. To show that $t^* < \min_j \pi/\omega_j$, we rely on a particular property of matrix Riccati differential equations S36: their solutions preserve the order generated by the cone of non-negative symmetric matrices, see [10]. More precisely, if $S_1(t)$ and $S_2(t)$ are solutions of S36, and if $S_1(0) \preceq S_2(0)$, then $S_1(t) \preceq S_2(t)$, for all $t \geq 0$ for which both solutions are defined. The partial order notation $S_1(t) \preceq S_2(t)$ means that the difference $S_2(t) - S_1(t)$ is a positive semi-definite matrix.

We apply this to equation S35 with $\tilde{S}_1(0) = \alpha_{\min} I_n$ and $\tilde{S}_2(0) = \tilde{S}(0)$, where we choose $\alpha_{\min}$ as the smallest eigenvalue of $\tilde{S}(0)$ (or equivalently, of $S(0) = S_0$, since $\tilde{S}(0) = V^T S_0 V$), so that clearly $\tilde{S}_1(0) \preceq \tilde{S}_2(0)$. Consequently, by the monotonicity property of system S35, it follows that $\tilde{S}_1(t) \preceq \tilde{S}(t)$, as long as both solutions are defined. We can calculate the blow-up time t_1^* of $\tilde{S}_1(t)$ explicitly, and then it follows that $t^* \leq t_1^*$, where t^* is the blow-up time of $\tilde{S}(t)$. Indeed, equations of system S35 decouple for an initial condition of the form $\alpha_{\min} I_n$, and the resulting scalar equations are scalar Riccati equations we have solved before. The blow-up time for $\tilde{S}_1(t)$ is given by:

$$t_1^* = \begin{cases} \min_{j=1,\ldots,k} \left(\frac{\pi}{2\omega_j} - \frac{\phi_j}{\omega_j} \right), & \text{if } \alpha_{\min} \leq 0 \\ \min_{j=1,\ldots,k} \left(\frac{1}{\alpha_{\min}}, \frac{\pi}{2\omega_j} - \frac{\phi_j}{\omega_j} \right), & \text{if } \alpha_{\min} > 0 \end{cases}.$$

with $\phi_j := \arctan\left(\frac{\alpha_{\min}}{\omega_j}\right) \in \left(-\frac{\pi}{2}, \frac{\pi}{2}\right)$. Notice that for all $j = 1, \ldots, k$, there holds that $\frac{\pi}{2\omega_j} - \frac{\phi_j}{\omega_j} < \frac{\pi}{\omega_j}$, because by definition, $\frac{\phi_j}{\omega_j} \in (-\frac{\pi}{2\omega_j}, \frac{\pi}{2\omega_j})$. Consequently,

$$\bar{t} \leq t^* \leq t_1^* < \min_{j=1,\ldots,k} \frac{\pi}{\omega_j},$$

which establishes S44. In other words, we have shown that the first item in assumption A is always satisfied.

The second item in assumption A may fail, but holds for generic initial conditions as we show next. For this we first point out that the derivative of each eigenvalue of $M(t)$ is a strictly decreasing function

in the interval $(0,\bar{t})$, independently of the value of the matrix $\tilde{S}_0$. Indeed, the derivative of eigenvalue $\lambda_j(t)$ of $M(t)$ equals (see [7]) :

$$\dot{\lambda}_j(t) = u_j(t)^T \dot{M}(t) u_j(t) = -u_j(t)^T \begin{pmatrix} 1/t^2 & 0 \\ 0 & \tilde{D}^2 s^{-2}(t) \end{pmatrix} u_j(t),$$

where $u_j(t)$ is the normalized eigenvector of $M(t)$ corresponding to $\lambda_j(t)$, and which is analytic in the considered interval. Since $\dot{M}(t)$ is negative definite in that interval, $\dot{\lambda}_j(t)$ is also negative and hence all eigenvalues of $M(t)$ are strictly decreasing functions of t in that interval. Suppose now that $M(t)$ has a multiple eigenvalue 0 at $t=\bar{t}$, then $M(\bar{t})$ is positive semi-definite since $\bar{t}$ is the first singular point of $M(t)$ and the eigenvalues are decreasing function of t. If we now choose a positive semi-definite $\Delta_{\tilde{S}_0}$ of nullity 1, such that $M(\bar{t}) + \Delta_{\tilde{S}_0}$ also has nullity 1, then the perturbed initial condition $(\tilde{S}_0)_p = \tilde{S}_0 - \Delta_{\tilde{S}_0}$ yields the perturbed solution $\tilde{S}_p(t)$ which can be factored as $Q_p(t)P_p^{-1}(t)$, and where $P_p(t) = \Delta(t)M_p(t)$ (note that $\Delta(t)$ remains the same as before the perturbation) for $M_p(t) = M(t) + \Delta_{\tilde{S}_0}$ which now has a single root at the same minimal value $\bar{t}$. To construct such a matrix $\Delta_{\tilde{S}_0}$ is simple since the only condition it needs to satisfy is that $M(\bar{t})$ and $\Delta_{\tilde{S}_0}$ have a common null vector. Those degrees of freedom show that the second item in assumption A is indeed generic.

Now that we have established that A generically holds, we show that S41 is satisfied also. The proof is by contradiction. Earlier, we have shown that $\bar{t} \leq t^*$. Thus, if we suppose that S41 fails, then necessarily $\bar{t} < t^*$. This implies that although $P(\bar{t})$ is singular, the solution $\tilde{S}(t)$ exists for $t=\bar{t}$. Our goal is to show that $\lim_{t\to\bar{t}} |\tilde{S}(t)|_F = +\infty$, which yields the desired contradiction (by the theory of ODE's).

We first claim the following:

$$\text{If } u \neq 0 \text{ and } P(\bar{t})u = 0, \text{ then } Q(\bar{t})u \neq 0. \tag{S45}$$

Indeed, if this were not the case, then there would exist some vector $\bar{u} \neq 0$ such that $P(\bar{t})\bar{u} = 0$ and $Q(\bar{t})\bar{u} = 0$. On the other hand, $P(t)$ and $Q(t)$ are components of the matrix product

$$\begin{pmatrix} P(t) \\ Q(t) \end{pmatrix} = \mathrm{e}^{tH} \begin{pmatrix} I_n \\ \tilde{S}_0 \end{pmatrix},$$

where H is defined in S39. Multiplying the latter in $t=\bar{t}$ by $\bar{u}$, and using the previous expression, it follows from the invertibility of $\mathrm{e}^{\bar{t}H}$ that $\bar{u} = 0$, a contradiction. This establishes S45.

In the previous section, we factored $P(t)$ as $P(t) = \Delta(t)M(t)$. Since $P(t)$ is non-singular on $[0,\bar{t})$, and singular at $\bar{t}$, it follows from S44 and the definition of $\Delta(t)$, that $M(t)$ is non-singular (and, in fact, positive definite as shown in the previous section) on $(0,\bar{t})$, and singular at $\bar{t}$ as well. Therefore, since $M(t)$ is symmetric and real-analytic, it follows from [7] that we can find a positive and real-analytic scalar function $\epsilon(t)$, and a real-analytic unit vector $u(t)$ such that:

$$M(t)u(t) = \epsilon(t)u(t),\ \epsilon(t) > 0 \text{ on } (0,\bar{t}),\ \epsilon(\bar{t}) = 0,\ |u(t)|_2 = 1,$$

where $|.|_2$ denotes the Euclidean norm. In particular, $M(\bar{t})u(\bar{t}) = 0$, and since $\Delta(\bar{t})$ is non-singular, it follows that $P(\bar{t})u(\bar{t}) = 0$. Then S45 implies that $Q(\bar{t})u(\bar{t}) \neq 0$. Define the real-analytic unit vector

$$v(t) = \frac{\Delta(t)u(t)}{|\Delta(t)u(t)|_2},\ \ t \in (0,\bar{t}),$$

and calculate

$$\begin{aligned} \lim_{t\to\bar{t}} |\tilde{S}(t)v(t)|_2 &= \lim_{t\to\bar{t}} |Q(t)P^{-1}(t)v(t)|_2 \\ &= \frac{|Q(\bar{t})u(\bar{t})|_2}{|\Delta(\bar{t})u(\bar{t})|_2} \lim_{t\to\bar{t}} \frac{1}{\epsilon(t)} = +\infty. \end{aligned}$$

Since for any real $n \times n$ matrix A, and for any unit vector x (i.e. $|x|_2 = 1$) holds that $|Ax|_2 \leq |A|_F$, it follows that $\lim_{t \to \bar{t}} |\tilde{S}(t)|_F = +\infty$. This yields the sought-after contradiction.

By combining Proposition 1 and the results in this subsection, we have proved the main result concerning the generic emergence of balance for solutions of system S28.

Theorem 5. *There exists a dense set of initial conditions X_0 in $\mathbb{R}^{n \times n}$ such that the corresponding solution $X(t)$ of S28 satisfies:*

$$\lim_{t \to \bar{t}} \frac{X(t)}{|X(t)|_F} = \frac{ww^T}{|ww^T|_F}.$$

with $w = e^{\bar{t}A_0} V \Delta^{-1}(\bar{t}) u$.

Proof. The set of initial conditions X_0 for which $A_0 \neq 0$ and assumption A holds is dense in $\mathbb{R}^{n \times n}$. □

Supporting References

1. Horn R, Johnson C (1985) Matrix Analysis. Cambridge: Cambridge University Press.

2. Harary F (1953) On the notion of balance of a signed graph. The Michigan Mathematical Journal 2: 143–146.

3. Cartwright D, Harary F (1956) Structural balance: a generalization of Heider's theory. Psychological Review 63: 277–293.

4. Kulakowski K, Gawronski P, Gronek P (2005) The Heider balance - a continuous approach. International Journal of Modern Physics C 16: 707–716.

5. Marvel SA, Kleinberg J, Kleinberg RD, Strogatz SH (2011) Continuous-time model of structural balance. Proceedings of the National Academy of Sciences of the United States of America 108: 1771–1776.

6. Rugh W (1996) Linear system theory. Upper Saddle River: Prentice Hall.

7. Kato T (1995) Perturbation Theory for Linear Operators. New York: Springer, 640 pp.

8. Bunse-Gerstner A, Byers R, Mehrmann V, Nichols NK (1991) Numerical computation of an analytic singular value decomposition of a matrix valued function. Numerische Mathematik 60: 1–39.

9. Still G (2001) How to split the eigenvalues of a one-parameter family of matrices. Optimization 49: 387–403.

10. De Leenheer P, Sontag ED (2004) A note on the monotonicity of matrix Riccati equations. Technical report, DIMACS.

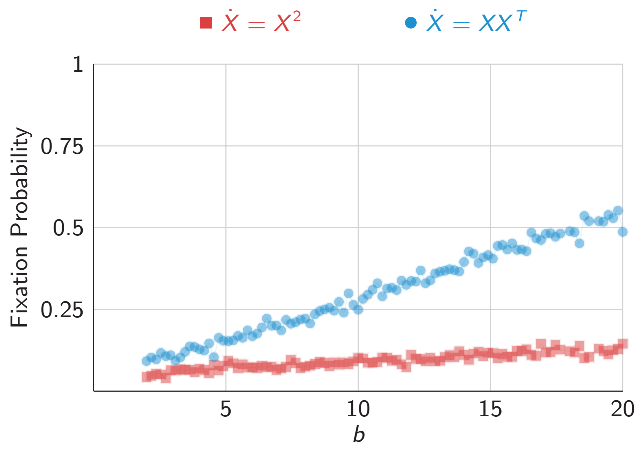

Figure S2. Results including type A, B and defectors.

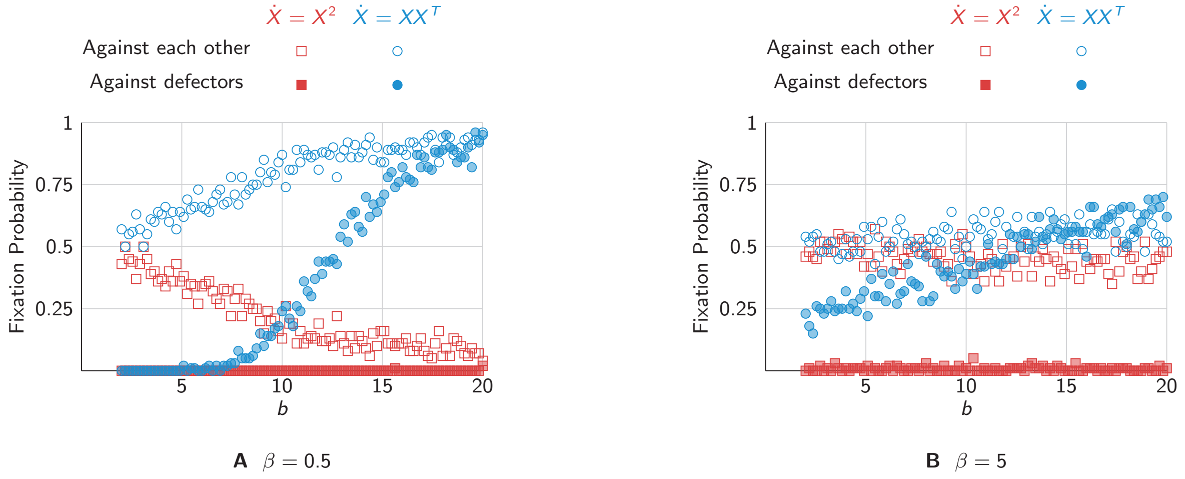

Figure S3. Results different intensities of selection.
